# Retarded germination of *Nicotiana tabacum* seeds following insertion of exogenous DNA mimics the seed persistent behavior

**DOI:** 10.1371/journal.pone.0187929

**Published:** 2017-12-07

**Authors:** Elisabetta Onelli, Alessandra Moscatelli, Assunta Gagliardi, Mauro Zaninelli, Luca Bini, Antonella Baldi, Marco Caccianiga, Serena Reggi, Luciana Rossi

**Affiliations:** 1 Department of Biosciences, University of Milano, Milan, Italy; 2 Laboratory of Functional Proteomic, Department of Life Science, University of Siena, Siena, Italy; 3 Department of Human Sciences and Quality of Life Promotion, Università Telematica San Raffaele Roma, Italy, Rome, Italy; 4 Department of Health, Animal Science and Food Safety, Università degli Studi di Milano, Milan, Italy; 5 Plantechno S.r.l., Vicomoscano, Cremona, Italy; United States Department of Agriculture, UNITED STATES

## Abstract

Tobacco seeds show a coat-imposed dormancy in which the seed envelope tissues (testa and endosperm) impose a physical constraint on the radicle protrusion. The germination-limiting process is represented by the endosperm rupture which is induced by cell-wall weakening. Transgenic tobacco seeds, obtained by insertion of exogenous genes codifying for seed-based oral vaccines (F18 and VT2eB), showed retarded germination with respect to the wild type and modified the expression of endogenous proteins. Morphological and proteomic analyses of wild type and transgenic seeds revealed new insights into factors influencing seed germination. Our data showed that the interference of exogenous DNA influences the germination rather than the dormancy release, by modifying the maturation process. Dry seeds of F18 and VT2eB transgenic lines accumulated a higher amount of reserve and stress–related proteins with respect to the wild type. Moreover, the storage proteins accumulated in tobacco F18 and VT2eB dry seeds have structural properties that do not enable the early limited proteolysis observed in the wild type. Morphological observations by electron and light microscopy revealed a retarded mobilization of the storage material from protein and lipid bodies in transgenic seeds, thus impairing water imbibition and embryo elongation. In addition, both F18 and VT2eB dry seeds are more rounded than the wild type. Both the morphological and biochemical characteristics of transgenic seeds mimic the seed persistent profile, in which their roundness enables them to be buried in the soil, while the higher content of storage material enables the hypocotyl to elongate more and the cotyledons to emerge.

## Introduction

In angiosperms, double fertilization enables the triploid endosperm to develop as reserve tissue, to supply nutrients for the embryo during germination and seedling [1]. The mechanisms involved in protein folding and mobilization upon seed imbibition regulate seed dormancy and the crucial steps of seedling emergence. Storage proteins are synthesized during seed maturation and are conserved in specialized tissues, such as in the endosperm and/or in the parenchyma of cotyledons [2]. The synthesis/storage and degradation of reserve proteins are tightly regulated. The way storage proteins are protected during seed maturation from uncontrolled proteolysis involves the deposit of reserve proteins into membrane-bounded organelles as vacuoles or protein bodies (PB) [3]. However, although the structural features of reserve proteins protect them from proteinases deposited in the same compartments, storage proteins such as legumins, albumins, some lectins and vicilins undergo limited proteolysis within the storage vacuoles [4, 5].

In addition to proteins, the endosperm accumulates lipids such as triacylglycerol, which are transformed into sucrose at the onset of seed germination [6]. On the other hand, proteomic characterization of the cress micropylar endosperm revealed the presence of proteins involved in protein folding, protein defense and stability [7]. This study also suggested that cress micropylar endosperm proteins may have a regulatory function as well as being the source of nutrition for the embryo [7].

Seed germination is defined by the emergence of the radicle through surrounding structures which in *Tobacco* correspond to the seed coat (testa) and micropylar endosperm [8, 9]. The dormancy break (which allows seeds to survive unfavorable conditions) occurs in dry tobacco seeds during after-ripening, a status characterized by physiological changes making the seeds ready for germination. After-ripening triggers active transcription and biochemical reactions which could lead to dormancy release [10–12]. It has also been shown that dormancy alleviation depends on non-enzymatic reactions associated with ROS (reactive oxygen species) which cause the formation of peroxy-lipids, carbonylated proteins, and oxidized mRNA. This selective oxidation of mRNA and proteins gradually occurs during storage, and influences the first few hours of imbibition leading to the maintenance or release of germination inhibition [13–17].

The uptake during imbibition leads to embryo cell elongation and radicle protrusion [18, 19]. When a radicle emerges from the micropylar endosperm, cells undergo cell cycle in order to form seedlings [20]. In *Arabidopsis*, germination and seedling growth have been shown to be accompanied by a significant reduction in stored metabolites, in parallel with the reactivation of metabolic pathways [21].

Edible vaccines (EVs) are represented by transgenic or xenogenic plants containing selected genes responsible for the expression of immunogenic proteins in their genome [22]. The fact that transgenic tobacco seeds obtained by insertion of genes codifying for the FedA (the main protein of the F18 adhesive fimbriae) and the B subunit of verocytotoxin from O138 verocytotoxic *E*.*coli* serotypes (VTEC) [23–25] displayed retarded germination with respect to the WT, prompted us to investigate the possible mechanisms regulating seed maturation and seedling.

EV has been known for years in plants, but how it may influence seed development and seed germination is hardly known since few careful investigations focused on this issue. The aim of this study was to evaluate the changes in morphological and proteomic traits induced by unintended effects of EV transgene integration into the plant genome in *N*. *tabacum* seeds, following a comparative approach with their near isogenic counterpart, and to correlate these changes with germination and seedling modifications.

We found that early germination stages of F18 and VT2eB transgenic seeds were delayed compared to the wild-type (WT). In addition, changes were also observed both in the shape of seeds and in the behavior of the reserve tissues. Light and transmission electron microscopy investigations revealed alterations in the embryo size and development. Modifications were also observed in the timing of reserve mobilization, which could also be related to the delay in seed germination. In addition, one dimensional (1D) gel electrophoresis showed that WT also differs from F18 and VT2eB seeds in terms of protein expression and that this difference is more acute in dry seeds compared with imbibed seeds. Data from 1D, two dimensional (2D) gel electrophoresis and mass spectrometry revealed that transgenic seeds accumulated a higher amount of reserve and chaperone proteins, together with proteins involved in seed dehydration and proteolytic enzymes.

The modulation of seed germination, related to changes in the expression of reserve and chaperone proteins and the ecological implications are discussed.

## Materials and methods

### Tobacco seeds

Transgenic *N*. *tabacum* lines *(cv*. *Xanthi)* transformed for the B subunit of VT2eB toxin (VT2eB) and for the major subunit FedA of the F18 adhesive fimbriae (F18) were considered (GenBank Accession number: VT2eB-X81417; F18-M61713). Briefly, the transgenic *N*. *tabacum* lines were obtained by agroinfection using pBIpGLOB binary vectors (DDBJ accession no. AX006477; S1 Fig) as described by Reggi et al.[26]. The encoding sequences, for VT2eB and for the major subunit FedA of the F18 adhesive fimbriae genes, were placed under the control of the soybean basic 7S globulin promoter for seed-specific expression [24]. Six homozygous transgenic lines of tobacco, three harboring respectively the Vt2e-B gene and three the FedA subunit of F18 fimbriae gene, were compared with the wild-type *N*. *tabacum (cv*. *Xanthi)* which was considered as the negative control (WT). All plants were grown in parallel and WT and transgenic seeds were collected in the same years from mother plants. The third generations (R3) of the experimental transgenic lines were randomly selected from a cultivation of a total of eighty F18 positive plants and one hundred and forty VT2eB positive plants, derived from the selected homozygous transgenic plants cultivated in a greenhouse in a period of 14 months.

### Evaluation of exogenous genes

Six independent lines (three transformed for VT2e-B and three transformed for F18 expression) of the third generations (R3) were evaluated for the presence of VT2e-B and F18 genes, respectively, by polymerase chain reaction (PCR) in two different experiments using each time about one thousand seeds. The reaction conditions were developed for a final volume of 50 μl, using 50 ng of template represented by genomic DNA purified from VT2eB and F18, respectively. For the VT2eB gene detection specific oligonucleotides were used (F:5’ atgaagaagatgtttatagcgg; R:3’ aacgggtccacttcaaatgatt). Thermal cycling was carried out using an initial denaturation step of 95°C for 5 min, followed by 30 cycles of denaturation at 95°C for 1 minute, annealing at 50°C for 1 minute and 20 seconds, and elongation at 72°C for 1 min 30 s. Cycling was completed by a final elongation step of 72°C for 5 minutes. For the F18 gene detection, specific oligonucleotides were used (F: 5’atgaaaagactagtgtttatttcttttg; R: 3’cgaatgcgccaatgaatgttcatt). Thermal cycling was carried out using an initial denaturation step of 95°C for 5 min, followed by 25 denaturation cycles at 95°C for 1 minute, annealing at 56°C for 1 minute and 20 sec, and elongation at 72°C for 1 min 30 s. Cycling was completed by a final elongation step of 72°C for 5 minutes.

### Seed germination

Wild type, F18 and VT2eB seeds of *N*. *tabacum* (L.) were sown in batches in soil and allowed to germinate under long-day conditions at 24°C ± 1 (14 h day/10 h night). The seeds were grown for three weeks and monitored every day.

Wild type, F18 and VT2eB seeds of *N*. *tabacum* (L.) were also sown on sterile wet paper and allowed to germinate in a culture room at 24°C under continuous light. Seeds were cultured for five days and the early stages of the germination process (3–5 days) were monitored in three different experiments (about 160 seeds were considered for each experiment), by a Leica light microscope DM RB, using a Leica N PLAN 2.5X objective. Images were collected using a Leica video camera MC 170 HD.

### Light and transmission electron microscopy

Seeds of WT, F18 and VT2eB were imbibed for 24 hours using distilled water. For fixation and embedding, both whole seeds and the separated embryo and seed coats were considered. Samples were fixed in 50mM Hepes, pH 7.4, 2% formaldehyde and 0.2% glutaraldehyde, overnight at 4°C and then repeatedly rinsed in 50mM Hepes, pH 7.4, dehydrated with increasing concentrations of methanol and embedded in LR white resin (Sigma). Semi-fine sections (2μm) and ultra-thin sections (80 nm), were obtained using a Reichert Jung Ultracut E microtome. Semi-fine sections were stained by 1% toluidine blue and observed with a Leica DMRB light microscope. Ultra-thin sections were stained with 3% uranyl-acetate and observed with an EFTEM LEO 912AB transmission electron microscope (Zeiss) working at 80 kV. Five imbibed seeds for 3 different embedding experiments were analyzed for EM and light microscopy for each sample. The observations were performed on longest embryos for WT and transgenic lines.

The size of the seeds and embryos were measured using ImageJ. The values were processed for statistical analysis (t-test) by Microsoft Excel.

### Seed protein extraction

Wild type, F18 and VT2eB seeds of *N*. *tabacum* were imbibed in distilled water for 24 hours at 4°C. Dry and hydrated wild type, F18 and Vte2B tobacco seeds were frozen in liquid nitrogen and ground to powder. The homogenate was resuspended using the extraction buffer (EB): 8 M urea, 40 mM Tris-HCl, 20 mM DTT, 2% Tween-20, 5 mM PMSF and incubated for 1 hour at 4°C, vortexing every 10 minutes to facilitate protein extraction. The homogenates were subsequently centrifuged at 15°C for 30 minutes at 18.000 *g* (13.000 rpm) in an ALC A21-C rotor. The resulting supernatants were collected and stored at -20°C as crude extracts. Aliquots of crude extracts were protein assayed (Bradford), using BSA as a standard protein.

### One- and two-dimensional electrophoresis

Proteins (20 μg/lane) were resolved in denaturing 15% polyacrylamide one-dimensional (1D) gels in a discontinuous buffer system following Laemmli [27]. MiniVe Vertical Electrophoresis System (GE Healthcare, USA) was used for 1D electrophoresis. Proteins were stained with Coomassie brilliant blue R250. 1D-electrophoresis were replicated five times.

2D electrophoresis was performed using the Immobiline-polyacrylamide system, as previously described [28, 29]. IEF was carried out on non-linear wide-range immobilized pH gradients (IPG) (pH 4–7; 18 cm long IPG strips; GE Healthcare, Uppsala, Sweden) and performed using the Ettan^™^ IPGphor system (GE Healthcare). Strips for analytical runs were rehydrated with 60 μg of protein in 350 μl of EB and 0.2% (v/v) carrier ampholyte for 1 h at 0 V and for 8 h at 30 V, at 16°C. The strips were then focused according to the following electrical conditions at 16°C: 200 V for 1 h, from 300 V to 3500 V in 30 min, 3500 V for 3 h, from 3500 V to 8000 V in 30 min, 8000 V for 3 h, 10.000 V until a total of 80.000 Vh was reached.

MS-preparative strips were rehydrated with 500 μg of protein in 350 μl of EB and 2% (v/v) carrier ampholyte, for 1 h at 0 V and overnight at 30 V, at 16°C. Then IEF was achieved setting the following voltage steps at 16°C: 200 V for 8 h, from 200 V to 3500 V in 2 h, 3500 V for 2 h, from 3500 V to 5000 V in 2 h, 5000 V for 3 h, from 5000 V to 8000 V in 1 h, 8000 V for 1 h, from 8000 V to 10.000 V in 1 h, 10.000 V until a total of 100.000 Vh was reached.

After IEF, strips were subjected to two equilibration steps: the first was for 12 min in 6 M urea, 30% (v/v) glycerol, 2% (w/v) SDS, 0.05 M Tris–HCl pH 6.8, 2% (w/v) DTE; and the second one was for 5 min in 6 M urea, 30% (v/v) glycerol, 2% (w/v) SDS, 0.05 M Tris–HCl pH 6.8, 2.5% (w/v) iodoacetamide, and bromophenol blue in trace.

The second dimension was carried out, at 10°C, on house-made 9–16% polyacrylamide linear gradient gels at 40 mA/gel constant current, until the dye front reached the bottom of the gel. Analytical and MS-preparative gels were stained with ammoniacal silver nitrate [30, 31] and with MS-compatible silver staining [32], respectively. They were then scanned using an ImageScanner III (GE Healthcare).

### Image analysis and statistics

Image analysis was performed on analytical 2D gels using ImageMaster 2-D Platinum v6.0 (GE Healthcare). For each condition tested, image analysis was performed on three different spot maps from three biological replicates for a total of nine analyzed gels. An intra-class quality and experimental control was performed by comparing the gels and then a differential inter-class analysis was performed to detect any statistically significant quantitative and qualitative differences. Based on a fold change of at least ±2 in relative volume (%V) ratio, and on statistically analysis as reported in the tables, differentially expressed proteins were detected among the three conditions examined.

### Protein identification by mass spectrometry

Protein identification was performed by peptide mass fingerprinting [33, 34] using an Ultraflex III MALDI-TOF/TOF mass spectrometer (Bruker Daltonics, Billerica, MA). Bands and spots of interest were manually excised, destained, as previously described [34, 35], and acetonitrile dehydrated. Before protein digestion, 1D gel-resolved proteins were reduced and alkylated as previously reported [35]. 1D and 2D-gel resolved proteins were rehydrated in trypsin solution (Sigma Aldrich, Italy), and in-gel protein digestion was performed by an overnight incubation at 37°C. A total of 1.25 μl of each protein digest was directly spotted onto the MALDI target and air-dried. Then 0.75 μl of the matrix solution (a saturated solution of alpha-cyano-4-hydroxycynnamic acid in 50% v/v acetonitrile and 0.5% v/v trifluoroacetic acid) was added to the dried samples and allowed to dry again.

Mass spectra were acquired using the above-mentioned mass spectrometer in reflector positive mode with a laser frequency set to 100 Hz. Spectra were analyzed by Flex Analysis v.3.0. Peptide mass fingerprinting (PMF) database searching was carried out in NCBIprot database set for *Viridiplantae* (Green Plants) using Mascot (Matrix Science Ltd., London, UK, http://www.matrixscience.com) with the following settings: experimental and theoretical peptide fingerprinting patterns with a Δmass less than 100 ppm, trypsin as the digestion enzyme with one missed cleavage allowed, carbamidomethylation of cysteine and oxidation of methionine as fixed and variable modifications, respectively. For protein identifications, the number of matched peptides, the extent of sequence coverage, and the probabilistic score were considered [36].

## Results and discussion

### Transgenic seeds showed changes in the timing of germination and in the behavior of reserve tissues

The presence of VT2eB and F18 genes in the two lines of transgenic seeds was assessed by PCR. Our results confirmed the stable integration of the two exogenous genes in R3 generation of both lines (S1 Fig).

Preliminary experiments of seed germination on soil revealed a significant delay of F18 and VT2eB seedlings compared to WT, in four independent experiments. Whereas the WT seeds exposed the photosynthetic cotyledons in 4 days, transgenic lines F18 and VT2eB only reached the same developmental stage after 7 and 8 days, respectively (S2 Fig).

**Fig 1:**
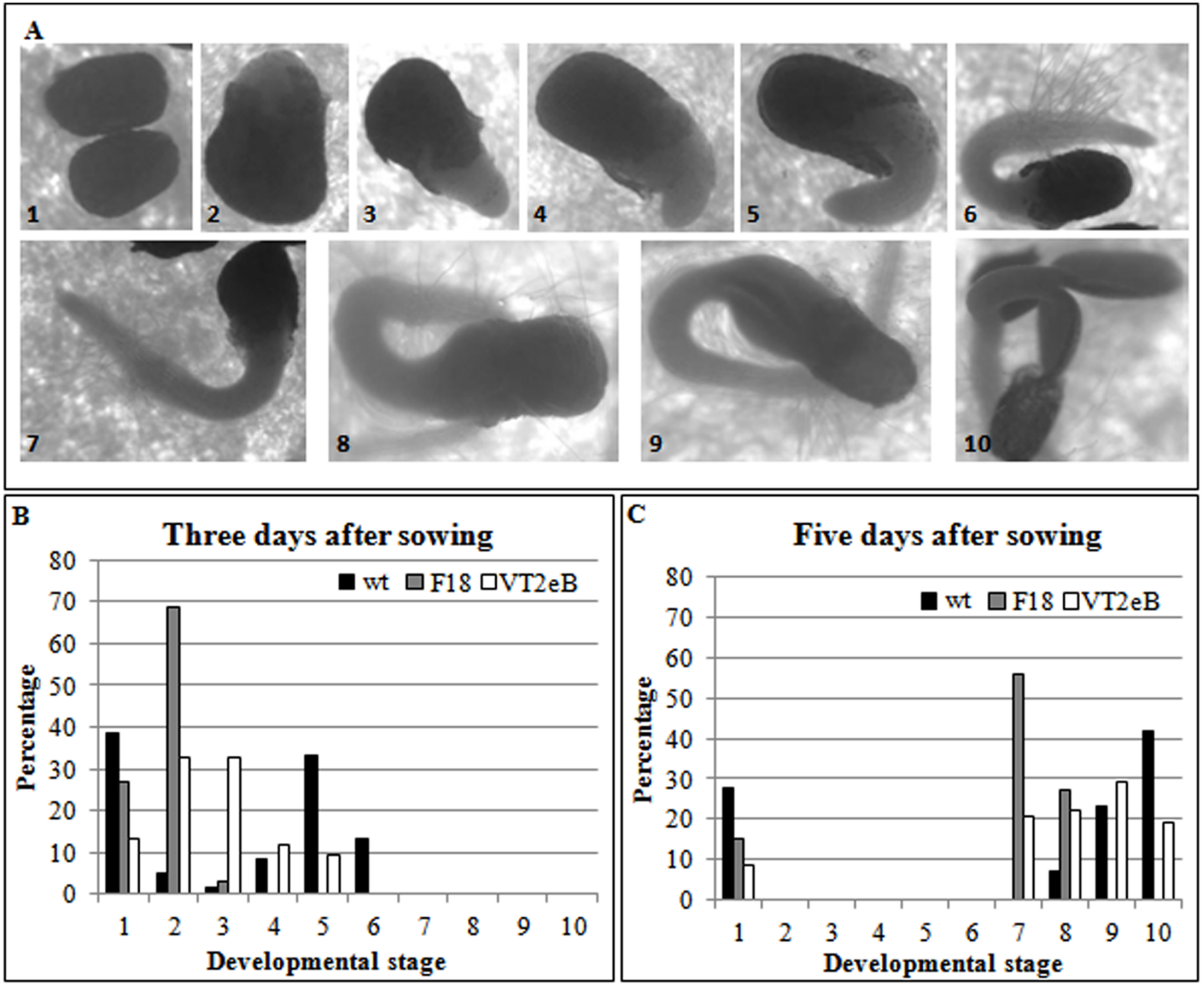
Time course experiments on tobacco seeds germination. **A** The observation of seeds germinated on wet filter paper for several days revealed ten different seed developmental stages. **B** The graphs described developmental behavior of WT and transgenic seeds three and five days after sowing. The analysis of data showed that the germination process was delayed in transgenic seeds and that VT2eB seedlings were less affected than F18 line.

To analyze the dynamics of seedlings in more detail, seeds of WT and transgenic lines germinated on wet filter paper, under controlled light and temperature conditions. Seeds were observed every 24 hours for five days. *Nicotiana* seed germination involved two steps: testa (seed coat) rupture followed by endosperm rupture [9]. Since the mycropilar endosperm was considered a germination constraint of *Solanaceae* seeds, the rupture of the mycropilar endosperm and the consequent emission of the radicle primordium was considered as the germination point in *N*. *tabacum* (visible germination, see Fig 1A, stage 3). Seven additional developmental stages were considered for the analysis (Fig 1A).

Germinating seeds of wild type and transgenic lines, observed at different time points after sowing (3 to 5 days), confirmed that the germination time of F18 and VT2eB strains was delayed with respect to WT, in two repeated experiments (Fig 1B and 1C). In the WT a considerable number of seeds had already developed roots three days after sowing (Fig 1A and 1B; about 50% considering stages 4, 5, 6). In contrast most of the F18 seeds underwent testa rupture but not micropylar endosperm rupture, while VT2eB seeds were mostly between stages 2 and 3, suggesting that these seeds are able to initiate the micropylar endosperm rupture (Fig 1A and 1B). The germination process, analyzed up to five days after seed sowing, showed that whereas most of the F18 germinating seeds were still in stage 7, in WT seeds, root and shoot meristems actively participated in seedling growth (Fig 1C). VT2eB seeds distributed in steps 7–10, thus supporting the idea that the VT2eB seedling was delayed in the early stages of the process but to a lesser extent than the F18 mutant.

**Fig 2:**
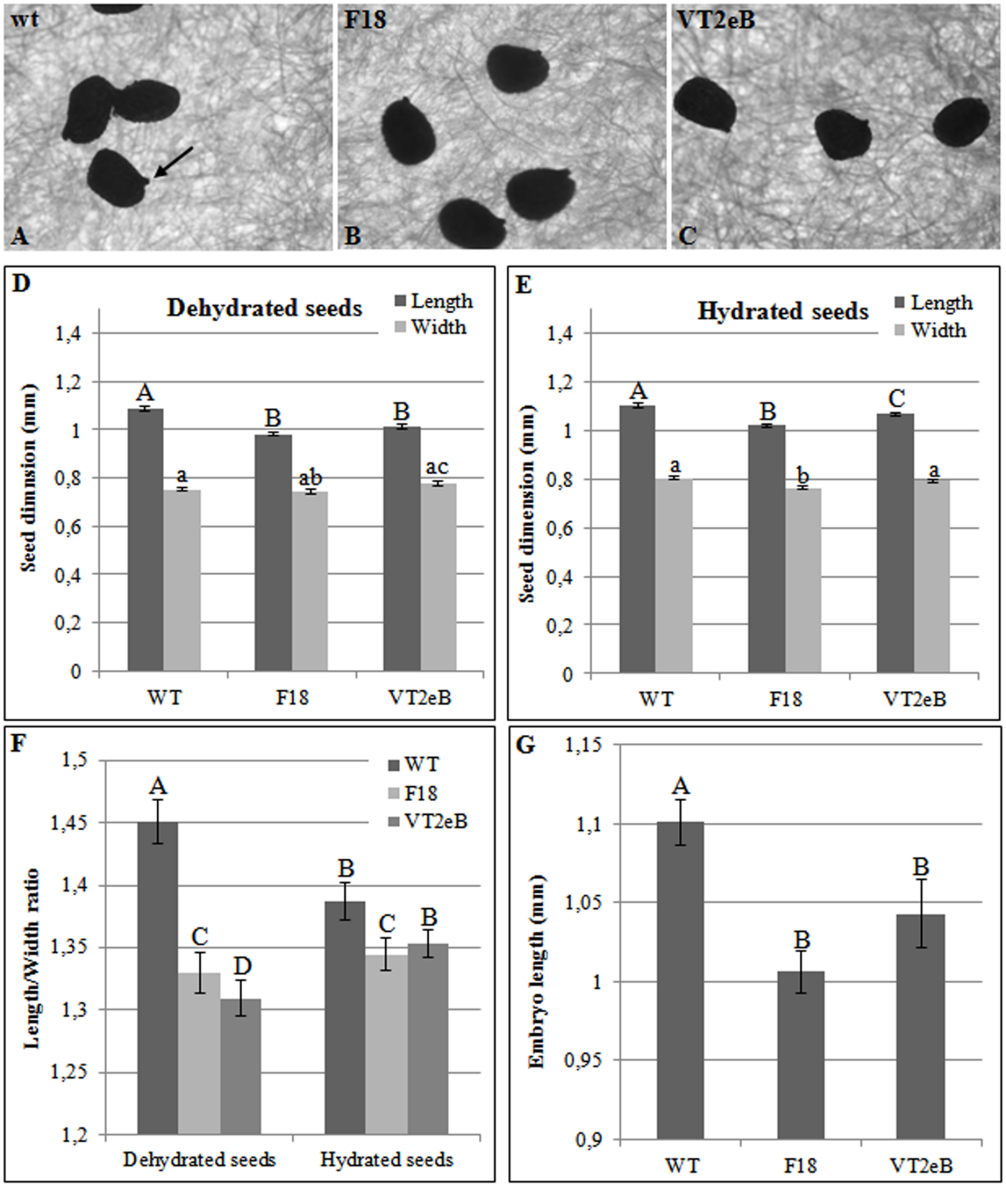
Morphological analysis of dry and hydrated seeds. **A**, **B**, **C** WT, F18 and VT2eB dry seeds (respectively) are egg shaped, with a prominent hilum at the germinating end (arrow). However, in transgenic lines, seeds were more rounded than WT. **D**, **E** The size of dry and imbibed seeds, calculated by measuring their major (length) and minor axis (width). The length of WT seeds was significantly higher than the transgenic seeds, while the width was less influenced by the exogenous DNA insertion, and significant differences were observed only between the two transgenic seed lines. **F** The seed shape was described considering the length/width ratio. Dehydrated seeds of F18 and VT2eB were significantly more rounded -than the WT, and VTe2B seeds were significantly more rounded than F18. During hydration, VT2eB seeds took on a similar shape to WT, while F18 seeds remained more rounded than the WT. **G** Measurement of the embryo length following the hydration process. WT embryos were significantly longer than the transgenic embryos.

To explain these differences, morphological analyses of WT, F18 and VT2eB seeds were carried out (Fig 2).

The sizes of dry and imbibed seeds (24 h in water) were calculated by measuring both their major (length) and minor axis (width) using ImageJ. Usually, dry seeds are egg shaped, with a prominent hilum at the germinating end (Fig 2A–2C, arrow). The insertion of exogenous DNA induced changes in both the seed size and shape. In dry WT, the seed was significantly longer than the transgenic seeds (Fig 2D: *p*<0.01), while the width was less influenced by the exogenous DNA insertion, and significant differences were observed only between the two transgenic seed lines (Fig 2D; *p*<0.01). Considering the length/width ratio as the value describing the shape, dehydrated seeds of F18 and VT2eB were significantly rounder than the WT (Fig 2B and 2F; *p*<0.01) and VTe2B seeds were significantly rounder, with respect to F18 (Fig 2C and 2F; *p*<0.01). The differences in seed size and shape were attenuated following the imbibition process (Fig 2E).

The water input accounts for a general increase in seed size; in particular the imbibed WT became rounder while the VTe2B seeds lost their round form compared to the dry seeds (Fig 2F; *p*<0.05). Conversely, the shape of the F18 seeds did not change during the imbibition (Fig 2F).

The seed size analyses also highlighted that a number of WT seeds (about 8%) showed the testa breaking and the growth of the radicle primordium as soon as 24 hours after imbibition (type 2, Fig 1). In contrast, this stage was not observed in the transgenic lines. In addition, once isolated from the seed coat, WT embryos appeared significantly longer than the transgenic embryos (Fig 2G; *p*<0.01 WT versus F18; *p*<0.05 WT versus VT2eB). Radicle protrusion depends on embryo expansion which is driven by water uptake. In this early phase of germination, cell proliferation does not occur [18, 37].

The differences observed in embryo elongation among WT and F18 /VT2eB seeds could be related to the modification of water uptake. Water uptake occurs in three phases involving a rapid initial imbibition (phase I), a subsequent slowdown (phase II) and a further uptake, starting late in phase II and continuing in phase III [18]. Phases I and II support the initial embryo elongation and testa rupture. The endosperm rupture occurs in phase III and is ABA and β-1,3 glucanase dependent [18]. After 24 h of imbibition, tobacco seeds were at the onset of phase II [18] and thus the embryo elongation observed in WT tobacco seeds was only due to hydration. In mutant seeds, this phase of water uptake appeared to be impaired. Since in oilseeds water is absorbed almost exclusively by carbohydrates and proteins [18], carbohydrates and/or storage proteins may have been altered in F18 and VT2eB mutants, thus triggering the reduced embryo elongation.

**Fig 3:**
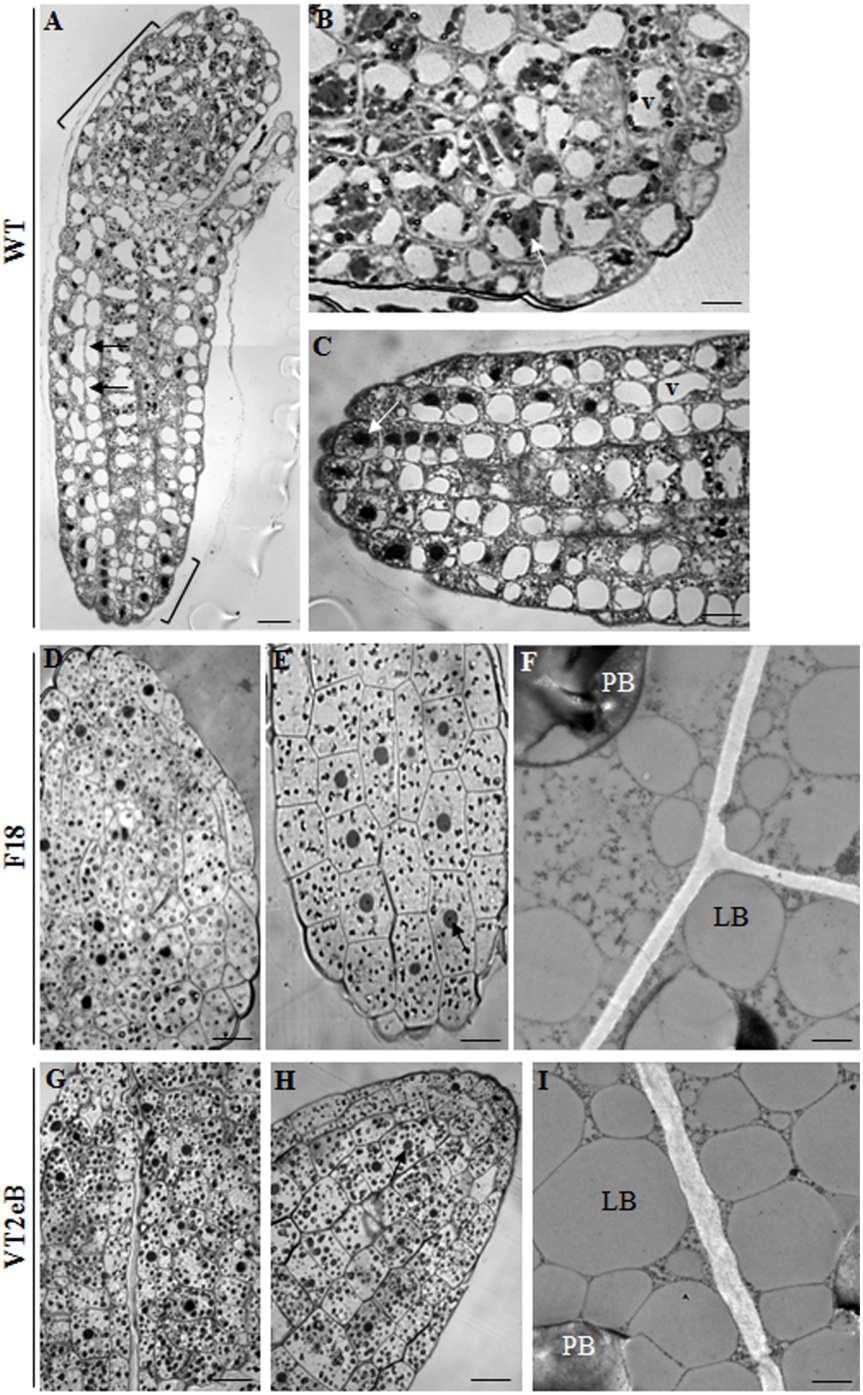
Anatomy of embryos by light and TEM microscopy. **A**-**C** WT embryo. Isodiametric cells were observed in the root meristems and in the cotyledons (square brackets). In the hypocotyl, cells appeared elongated along the major axis (black arrows), suggesting that the differentiation processes had already occurred. Most of the embryo cells showed a dense cytoplasm with prominent nuclei (white arrows), a large central vacuole (v), and the lack of storage vacuoles. This suggests that the reserve material was already mobilized, and embryo cells undergo root growth. Scale bar: 10 μm. **D, E** In F18 seeds, all the embryo cells appeared isodiametric, with evident nuclei (arrows), apparently without large vacuoles and with dark bodies ascribable to protein bodies (PBs). Scale bar: 10 μm. **F** TEM analyses showed the presence of both PBs and lipid bodies (LPs) in embryo cells. Scale bar: 300 nm. **G, H** In VT2eB seeds, all the embryo cells appeared isodiametric, with evident nuclei (arrows) and appeared not to have large vacuoles. There were more dark bodies ascribable to PB than in the F18 embryo. Scale bar: 10 μm. **i** TEM analyses showed the presence of BPs and LPs in embryo cells. Scale bar: 300 nm.

**Fig 4:**
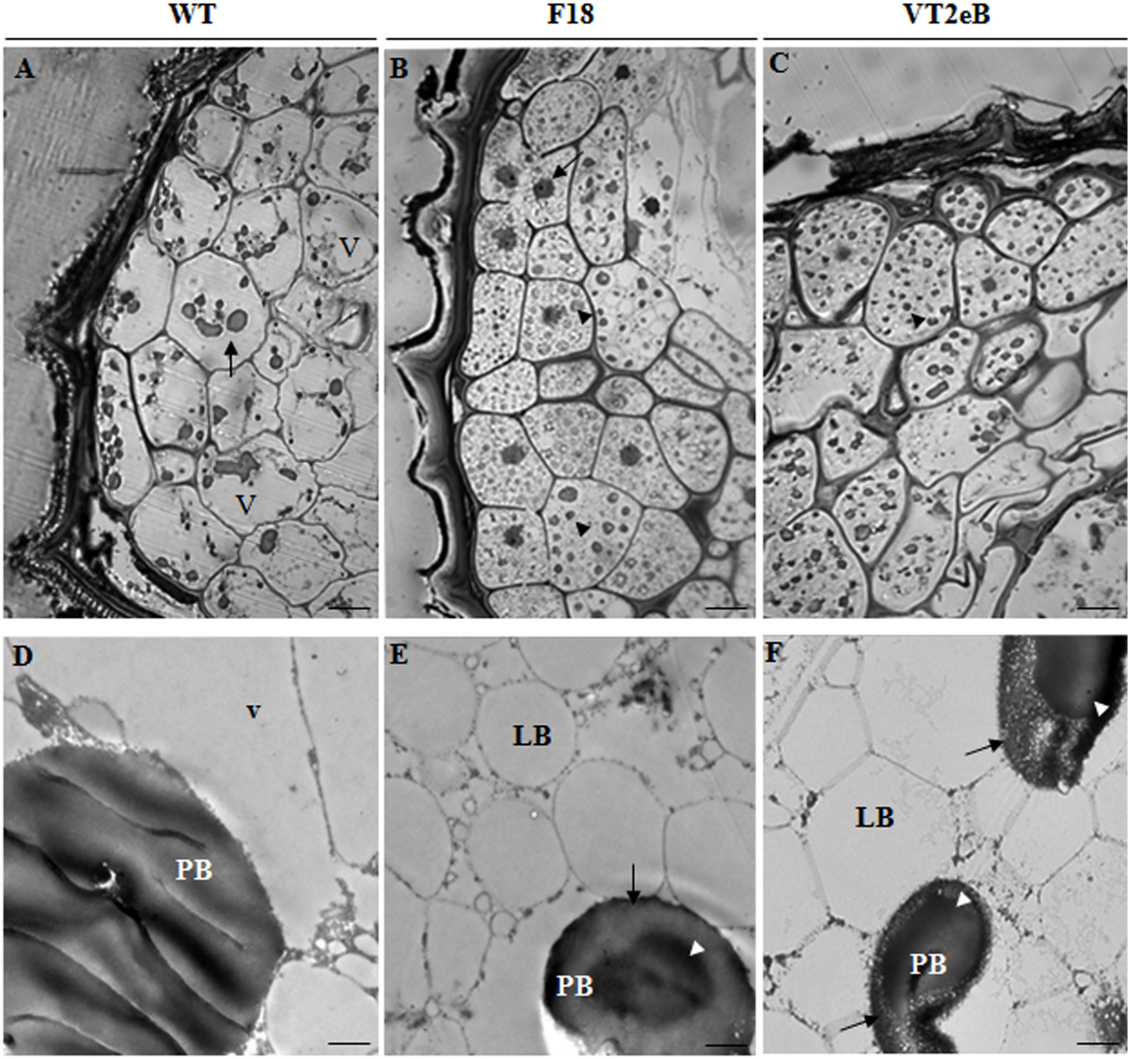
Anatomy of the endosperm by light and TEM microscopy. **A** In the WT endosperm cells, under an light microscope cells appeared isodiametric, with large vacuoles (v) and with dark bodies ascribable to PBs (arrow). Scale bar: 10 μm. **B, C** In transgenic seeds endosperm cells showed prominent nuclei (arrow) and storage material (arrowheads). Large vacuole were not observed. Scale bar: 10 μm. **D-F** TEM analyses showed that in WT seeds the storage material was partially mobilized: PBs were still observed in endosperm cells, while LBs were no longer present. Large vacuoles were observed within the cells (v). In transgenic seeds both PBs and PLs were present. In WT seeds, PBs appeared to contain uniform, amorphous electrondense material, the PBs of transgenic endosperm cells showed electron-dense structures surrounded by a lower dense matrix (white arrowheads and black arrows, respectively). Scale bar: 300 nm.

To investigate the correlations between differences in the size and shape of the seeds and their anatomy, the endosperm and the isolated embryos were studied by light microscopy after 24h of water imbibition (Fig 3, embryo, and Fig 4, endosperm).

In order to facilitate the embedding, the seed coat was removed from both the WT and the transgenic lines. Interestingly, in transgenic seeds, both the embryo and the endosperm cells appeared isodiametric, with evident nuclei (Figs 3E, 3H and 4B; arrows), apparently without large vacuoles and with dark bodies which are present in higher numbers in VT2eB seeds (Figs 3D, 3E, 3G, 3H, 4B and 4C; arrowheads). TEM analyses of mutant embryos showed that dark bodies were indeed protein bodies (PBs), which appeared to be filled with electron-dense material (Fig 4F and 4I). These PBs are surrounded by numerous vacuoles ascribable to lipid bodies (LBs; Fig 4F and 4I). In the WT embryos, isodiametric cells were confined in the apex of the root (meristematic cells) and in the cotyledons (Fig 3A; square brackets), while the hypocotyl cells appeared elongated along their major axis (Fig 3A, arrows), suggesting that both the distension (in accordance with water uptake dependent elongation) and the differentiation arose in WT embryos, whereas the same processes were retarded in transgenic seeds. In addition, in the WT, most of the cells revealed a lack of storage vacuoles (both PBs and LBs; Fig 3B and 3C) and showed a dense cytoplasm with prominent nuclei (Fig 3B and 3C, white arrows) and a large central vacuole (Fig 3A–3C). This thus suggested that the storage material supporting germination and early seedling growth had already been mobilized and that embryo cells had undergone root growth.

The analysis of the endosperm cells by TEM confirmed that the storage material comprised both PBs (Fig 4, PBs) and LBs (Fig 4, LBs), and that in the WT seeds, the storage material had partially mobilized already after 24 hours of water imbibition, as LBs were no longer present. It is possible that lipids were the first to be mobilized, as an energetic source to support embryo growth, as some PBs could still be observed in endosperm cells (Fig 4A and 4D; arrow). Ultrastructural observations also revealed changes in the behavior of PBs. Whereas the PBs of WT seeds appeared as uniform, amorphous electron-dense material, the PBs of transgenic endosperm cells showed a core electron-dense structure surrounded by a lower dense matrix (Fig 4; white arrowheads and black arrows, respectively). The differences in PB ultrastructure in WT, compared with F18 and VT2eB suggested that different processes of protein folding occurred in order to package the storage proteins in the PBs [38]. The accumulation of misfolded proteins might affect the packaging and the accessibility of proteins to the processing enzymes, leading to changes in resource availability (see below in 2D-elecrophoretic analyses).

The disappearance of LBs in the WT endosperm cells, following water imbibition, suggested that these materials were mobilized early during seed germination compared to F18 and VT2eB. It has been shown that significant amounts of triacylglycerol accumulated in the endosperm cells of *Arabidopsis* seeds and that the carbohydrates derived from this lipid are required for embryo development [6]. In addition, in tobacco seeds, it has been shown that oil mobilization occurs during phase II of water uptake [18] which we also observed in WT seeds, since most LBs disappeared from the endosperm and embryo cells (Figs 3A–3C, 4A and 4D).

Therefore, in transgenic seeds the delay in the mobilization of reserve materials could reflect the hydration modification on which the use of the reserve material depends or it could be related to the accumulation of misfolded proteins during seed maturation. The observation of WT and transgenic dry seed morphology suggested that the delay in the early stages of germination in transgenic lines could be related to changes in seed maturation and after-ripening processes, which in turn enable the appropriate seed imbibition and germination.

The relationships between seed shape and delayed germination have been observed in many ecological studies dealing with seed persistence and dormancy [reviewed in 39]. Thompson et al. [40] found in 97 species of British flora that small and rounded seeds tend to persist in soil longer than elongated and/or flattened seeds. This pattern was confirmed for floras from different continents and ecological contexts [41–43] although with some exceptions for Australian and New Zealand flora [44, 45]. A highly significant relationship between seed morphology and the level of dormancy was observed for weed species by Gardarin and Colbach [46]. Large and elongated seeds may experience selection for a faster germination in order to avoid predation, while small and rounded seeds can be more easily buried in soil and delay germination. A negative correlation has also been reported between spherical seed and germination rate [47]. Seed shape has been found to be related to specific gene controlling hormone synthesis, metabolism or signaling pathways [48] and a relationship has been observed between the earliness of germination and the seed lipid content [46].

In addition, proteomic analyses reported in this study (see below) showed for the first time a relationship between seed persistent syndrome and storage protein content and processing. Our results suggest common mechanisms underpinning seed morphology and germination mechanisms.

### Protein analysis by 1D- and 2D-gel electrophoresis

**Fig 5:**
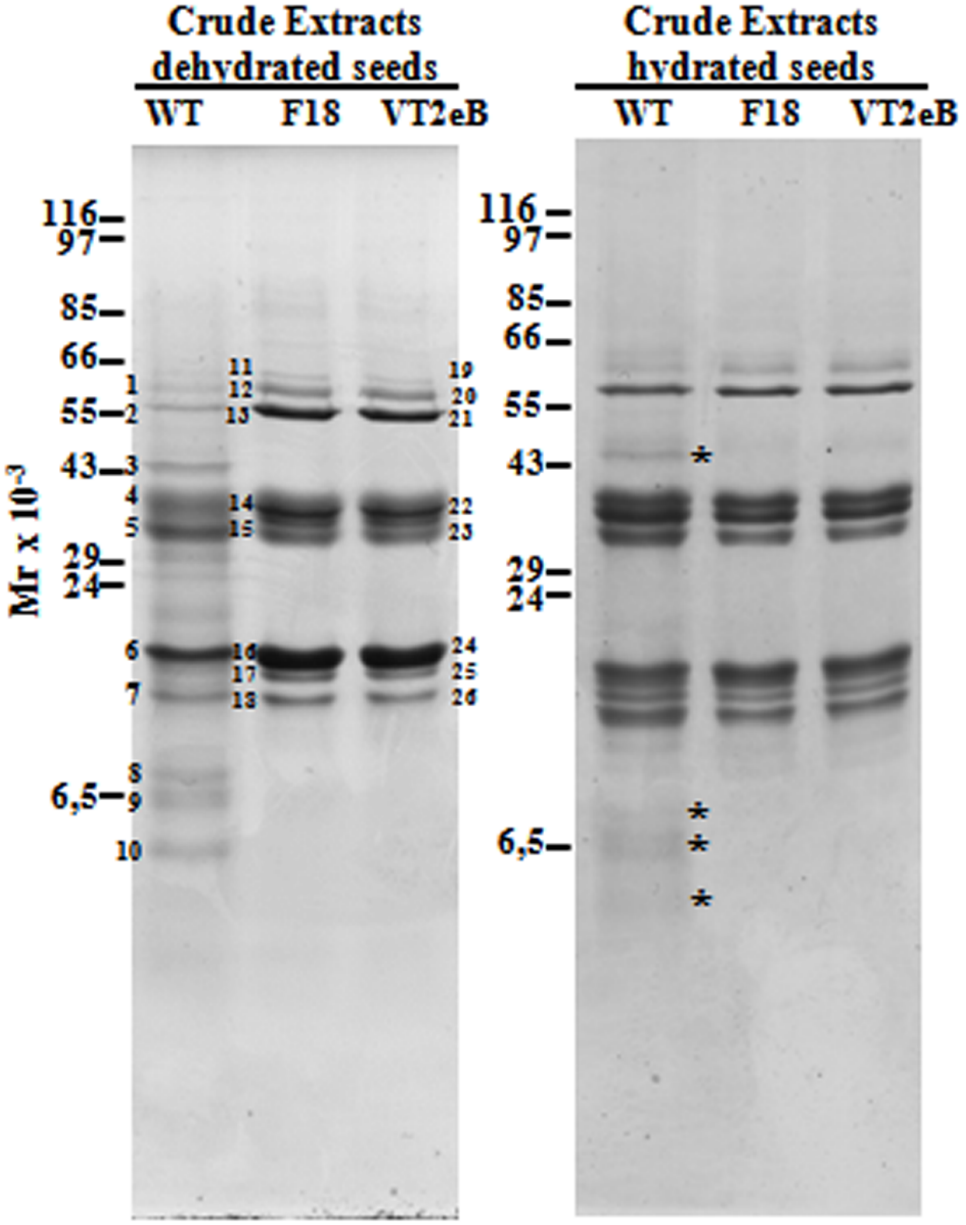
1-D gel electrophoresis of dry and hydrated seeds crude extracts. Qualitative differences in the polypeptidic profile was observed: while the intensity of some polypeptides seemed to be equivalent in the WT and in transgenic seeds, others increased in transgenic lines (polypeptides 11–13, 14–15 and 16–18 in F18 dry seed and 19–21, 22–23, 24–26 in VT2eB dry seed). On the other hand, low molecular weight polypeptides were present only in WT seeds (bands 8–10 in dry seed). In hydrated seeds the difference in the polypeptidic profile was less pronounced, and only polypeptides with a molecular mass of 43 kDa, and comprised between 6.5 and 14 kDa were observed in the WT (asterisks). In moist WT seeds, the polypeptides corresponding to band 2 and those corresponding to bands 6–7 had a higher intensity than the dry WT seeds. The band numbers correspond to polypeptides identified by MALDI TOF/TOF MS analysis showed in Table 1. The band numbers of the enhanced polypeptides are in bold.

To investigate the molecular basis of the differences observed by light and electron microscopy and to reveal whether modifications in transgenic lines were ascribable to alterations in seed maturation or germination, the protein profile of WT dry seeds was compared with that of F18 and VTe2B transgenic lines. One dimensional gel electrophoresis revealed qualitative differences in the protein profile. While the intensity of some polypeptides seemed to be the same in the two samples, others increased in transgenic seeds (Fig 5).

Polypeptides with a molecular mass of 55 kDa and about 20 kDa were enhanced in F18 and VTe2B with respect to the WT (Fig 5, compare bands 11–13 and 19–21 with bands 1–2; compare bands 16 and 24 with the band 6; compare bands 18 and 26 with the band 7). In addition, no polypeptide with a molecular weight ranging around 14 kDa in the F18 and VT2eB, was observed in the WT seeds (Fig 5, bands 17 and 25). On the other hand, one polypeptide with a molecular mass of 43 kDa (Fig 5, band 3) and three polypeptides with molecular mass of between 6.5 and 14 kDa (Fig 5, bands 8–10) were observed in WT and were not present in F18 and VT2eB dry seeds.

In hydrated seeds, the difference in the protein profile was less pronounced (Fig 5), since only the polypeptide with a molecular mass of 43 kDa, and three comprised between 6.5 and 14 kDa were present in the WT and were not observed in the F18 and in the VT2eB transgenic seeds (Fig 5, asterisks). The presence of low molecular weight polypeptides only in WT seeds suggested that after 24h of imbibition, the protein degradation in mutant seeds had not started, thus confirming the delay in storage mobilization. In *Arabidopsis*, the embryo elongation and subsequent seedling growth were associated with the increase in proteins involved with RNA translation, cell wall modification and protein degradation, which occur in imbibed seeds [18, 49]. The lack of protein degradation in mutant seeds suggested that some processes supporting metabolic events may be altered. In addition, biochemical analyses showed a lower number of reserve proteins in WT compared to the F18 and VT2eB tobacco dry seeds (Fig 5), thus the process allowing storage protein accumulation also appeared to be altered in mutant seeds.

In moist WT seeds, the polypeptides corresponding to band 2 and between bands 6–7 (Fig 5) showed a higher intensity compared to dry seeds, suggesting *de novo* synthesis of these proteins during seed hydration. Proteomic analyses of *Arabidopsis* seeds during germination revealed that the accumulation of cruciferin (the main seed storage protein in *Arabidopsis*) occurred by *de novo* synthesis during after-ripening in order to provide an additional source of amino acids and nitrogen to seedlings [50]. These data suggest a modification in the processes controlling the accumulation of storage proteins and the storage mobilization, thus contributing to the delay in F18 and VT2eB seed germination.

**Fig 6:**
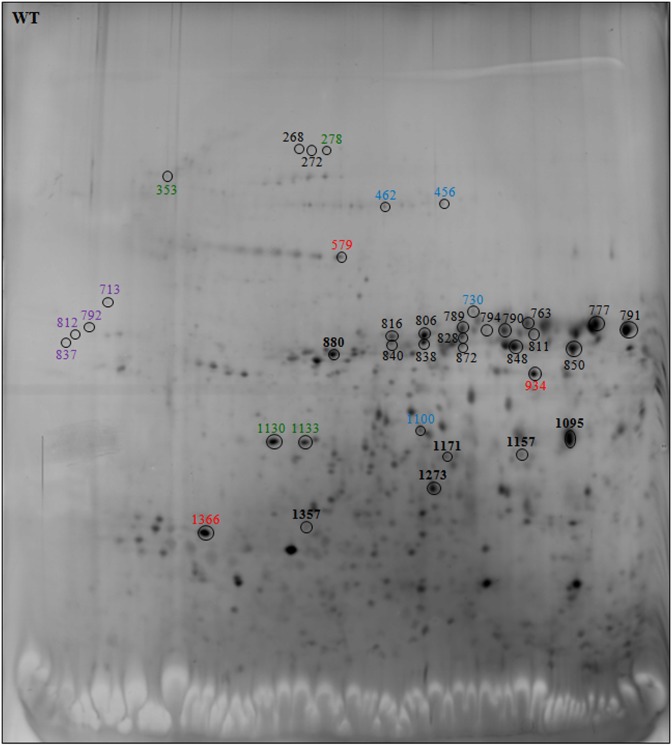
2D gel electrophoresis of WT seeds. Polypeptides that changed in transgenic lines with respect to the WT were highlighted with a circle. The number of spots corresponds to polypeptides identified by MALDI TOF/TOF MS analysis. Storage proteins are highlighted in black, Chaperone proteins in green, LEA proteins in violet, enzymes in blue, and other proteins in red.

**Fig 7:**
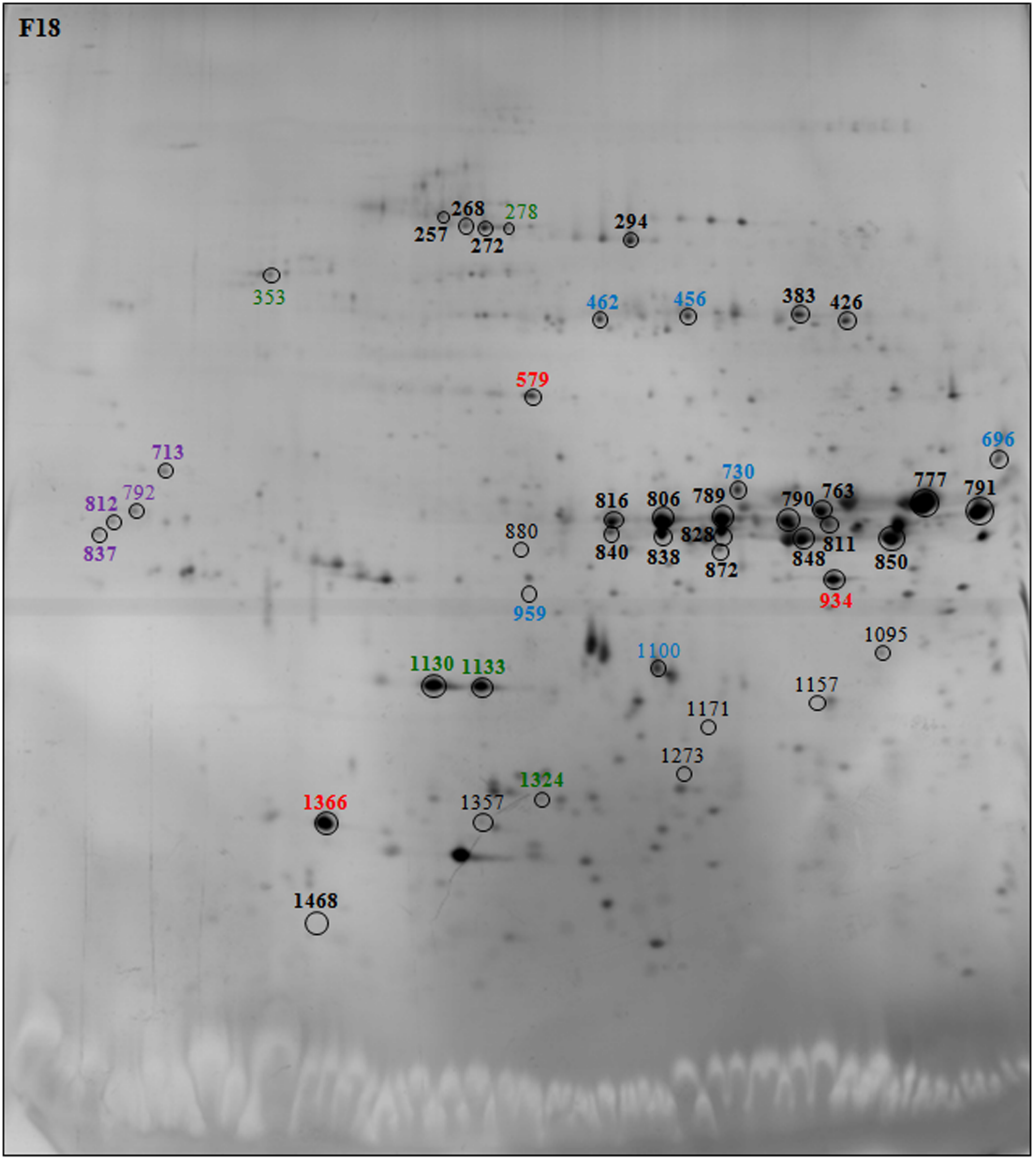
2D gel electrophoresis of F18 seeds. Polypeptides changed in transgenic line with respect to the WT were evidenced by circle. The number of spots correspond to polypeptides identified by MALDI TOF/TOF MS analysis. Storage proteins are highlighted in black, Chaperone proteins in green, LEA proteins in violet, enzymes in blue, and other proteins in red. Spot numbers of the enhanced polypeptides compared to the WT are in bold.

**Fig 8:**
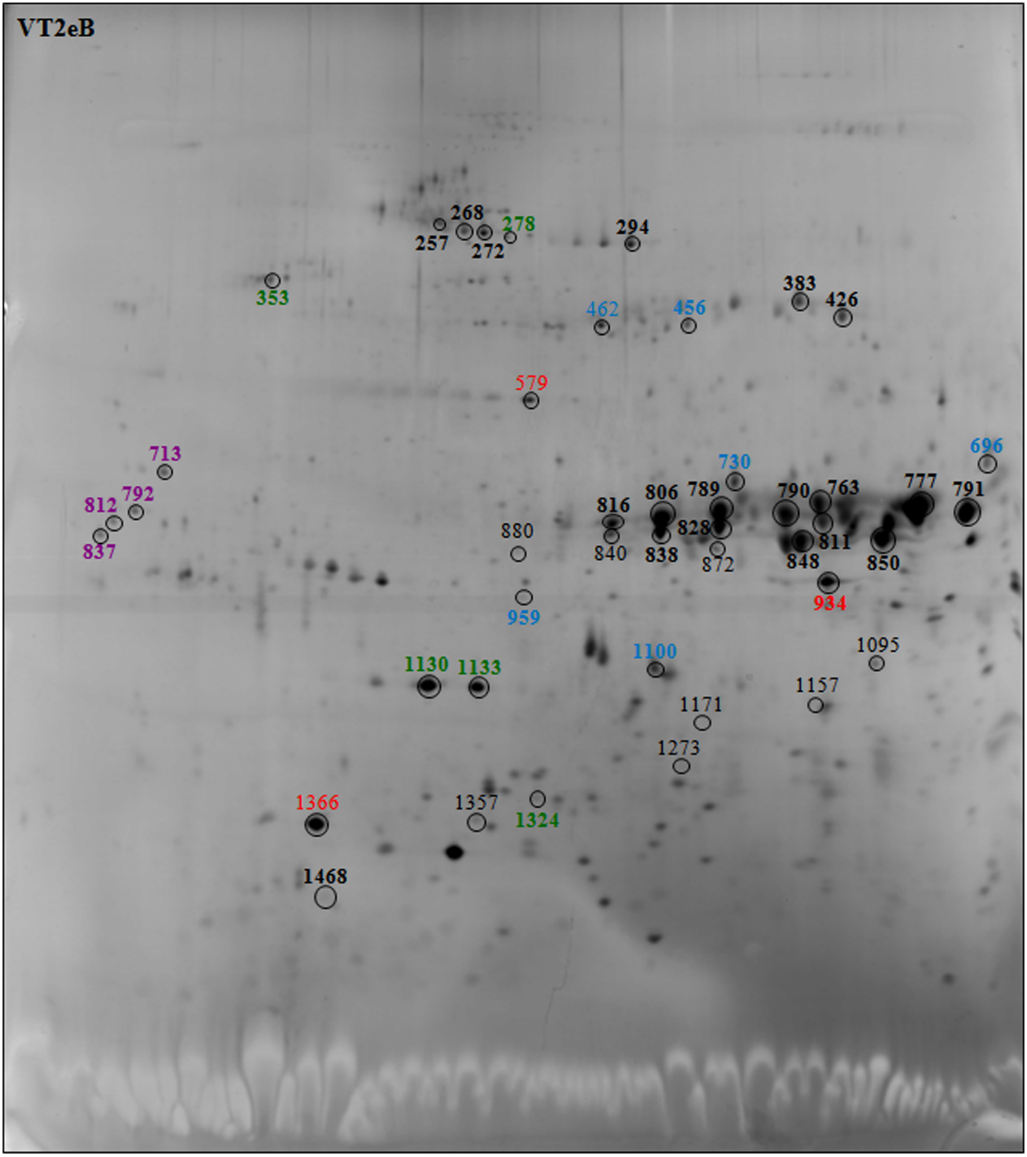
2D gel electrophoresis of VT2eB seeds. Polypeptides changed in transgenic line withrespect to the WT were evidenced by circle. The number of spots correspond to polypeptides identified by MALDI TOF/TOF MS analysis showed in Table 2. Storage proteins were evidenced in black, Chaperone proteins in green, LEA proteins in violet, enzymes in blue, and other proteins in red. Spot numbers of the enhanced polypeptides compared to the WT are in bold.

During seed germination, *de novo* transcription is not required and early germination events depend on the mRNA and protein stored during seed maturation, highlighting the hypothesis that germination has already been prepared during maturation [19, 49, 51]. To investigate whether the delay in early seed germination observed following exogenous DNA insertion could be due to changes in maturation rather than germination, the differences in the protein expression of WT, F18 and VT2eB dry seed crude extracts were analyzed in greater detail by 2D gel electrophoresis. The 2D gel analysis showed that a number of spots significantly varied in the transgenic seeds with respect to the WT (Fig 6 for WT and Figs 7 and 8 for F18 and VT2eB, respectively; varied spots were colored).

This thus suggests that the insertion of exogenous genes induced changes in the protein expression before the seed activation, and that these variations could lead to changes in seed imbibition and in the timing of reserve mobilization.

### Protein identification

To unravel the possible role of differentially expressed proteins in regulating the germination time of transgenic seeds with respect to the WT, polypeptides from dry seeds were excised from 1D and 2D gels and subjected to MALDI TOF/TOF MS analysis.

Mass spectrometry identified most of the polypeptides separated by 1D gel (Table 1), showing that enhanced polypeptides in the transgenic seeds are in fact storage proteins, belonging to the vicilin, legumin and globulin protein families (band numbers of the enhanced polypeptides are in bold), suggesting that a higher amount of reserve proteins accumulated in F18 and VT2eB dry seeds with respect to the WT. However, the polypeptides corresponding to bands 4 and 5 of the WT, comigrating with bands 14–15 and 22–23 of the F18 and VT2eB lines (Fig 5), respectively, were identified both as storage proteins and as centromere-associated protein E-like isoform X2, a mitotic kinesin that is required for the stable microtubule capture at kinetochores [52, 53]. However, in the F18 mutant, 14–15 bands showed only storage proteins and there was no centromere-associated protein E-like isoform X2. On the other hand, in VT2eB seeds this kinesin was only present in the 23 band (Table 1).

**Table 1:** 1D gel-resolved seed proteins identified by MALDI-TOF/TOF MS.

Band N.	Protein description	Accession number	Organism	Mascot search results		
				N. of matched peptides	Sequence coverage (%)	Score
***Wild type***						
1	**PREDICTED: vicilin-like antimicrobial peptides 2–2**	**gi|697094707**	***N*. *tomentosiformis***	14/37	26	119
	**PREDICTED: vicilin-like antimicrobial peptides 2–3**	**gi|698426490**	***N*. *sylvestris***	13/37	24	106
2	**PREDICTED: vicilin-like antimicrobial peptides 2–3**	**gi|698557844**	***N*. *sylvestris***	22/44	26	176
	**PREDICTED: vicilin-like antimicrobial peptides 2–3 isoform X2**	**gi|697173575**	***N*. *tomentosiformis***	21/44	26	164
**3**	**PREDICTED: vicilin-like antimicrobial peptides 2–3 isoform X2**	**gi|697173575**	***N*. *tomentosiformis***	24/70	29	139
	**PREDICTED: vicilin-like antimicrobial peptides 2–3**	**gi|698557844**	***N*. *sylvestris***	23/70	25 C-fragment	125
4	**PREDICTED: legumin B-like**	**gi|698534493**	***N*. *sylvestris***	18/102	33	107
	**PREDICTED: 11S globulin subunit beta-like**	**gi|697189071**	***N*. *tomentosiformis***	17/102	33	94
	**PREDICTED: centromere-associated protein E-like isoform X2**	**gi|721694598**	***B*. *distachyon***	30/102	19	79
5	**PREDICTED: legumin A-like**	**gi|697139891**	***N*. *tomentosiformis***	18/81	39	122
	**PREDICTED: legumin A-like**	**gi|698529732**	***N*. *sylvestris***	17/81	38	111
	**PREDICTED: centromere-associated protein E-like isoform X2**	**gi|721694598**	***B*. *distachyon***	26/81	19	94
6	**PREDICTED: legumin A-like**	**gi|697139896**	***N*. *tomentosiformis***	18/75	35	127
	**PREDICTED: 11S globulin seed storage protein 2-like**	**gi|697139889**	***N*. *tomentosiformis***	18/75	33	73
7	**PREDICTED: legumin B-like**	**gi|698534493**	***N*. *sylvestris***	18/91	31 C-fragment	85
**8**	**PREDICTED: legumin B-like**	**gi|698534493**	***N*. *sylvestris***	15/73	23	93
	**PREDICTED: legumin A-like**	**gi|698517368**	***N*. *sylvestris***	14/73	24 C-fragment	77
**9**	**PREDICTED: legumin B-like**	**gi|698534493**	***N*. *sylvestris***	17/95	34	92
	**PREDICTED: 11S globulin subunit beta-like**	**gi|697189071**	***N*. *tomentosiformis***	16/95	32	82
**10**	**PREDICTED: vicilin-like antimicrobial peptides 2–3**	**gi|698557844**	***N*. *sylvestris***	22/89	24	103
***F18***						
**11**	**PREDICTED: legumin A-like**	**gi|698517368**	***N*. *sylvestris***	12/39	26	103
**12**	**PREDICTED: vicilin-like antimicrobial peptides 2–3**	**gi|698426490**	***N*. *sylvestris***	22/69	42	163
**13**	**PREDICTED: vicilin-like antimicrobial peptides 2–3**	**gi|698557844**	***N*. *sylvestris***	31/64	34	223
**14**	**PREDICTED: 11S globulin seed storage protein 2-like**	**gi|697139889**	***N*. *tomentosiformis***	17/66	32	86
	**PREDICTED: legumin B-like**	**gi|698534493**	***N*. *sylvestris***	14/66	24	82
	**PREDICTED: 11S globulin subunit beta-like**	**gi|697189071**	***N*. *tomentosiformis***	14/66	24	82
**15**	**PREDICTED: legumin A-like**	**gi|697139891**	***N*. *tomentosiformis***	12/41	25 N-fragment	103
**16**	**PREDICTED: 11S globulin seed storage protein 2-like**	**gi|697139898**	***N*. *tomentosiformis***	14/60	68	98
**17**	**PREDICTED: 11S globulin seed storage protein 2-like**	**gi|697139898**	***N*. *tomentosiformis***	13/49	72	108
**18**	**PREDICTED: legumin B-like**	**gi|697151558**	***N*. *tomentosiformis***	15/54	22 C-fragment	92
***VT2eB***						
**19**	**PREDICTED: vicilin-like antimicrobial peptides 2–2**	**gi|697094707**	***N*. *tomentosiformis***	10/25	25	88
**20**	**PREDICTED: vicilin-like antimicrobial peptides 2–3**	**gi|698426490**	***N*. *sylvestris***	19/44	35	165
**21**	**PREDICTED: vicilin-like antimicrobial peptides 2–3**	**gi|698557844**	***N*. *sylvestris***	30/62	32 C-fragment	212
**22**	**PREDICTED: legumin B-like**	**gi|698534493**	***N*. *sylvestris***	15/73	25	98
	**PREDICTED: 11S globulin subunit beta-like**	**gi|697189071**	***N*. *tomentosiformis***	14/73	24	86
**23**	**PREDICTED: legumin A-like**	**gi|697139896**	***N*. *tomentosiformis***	12/42	27 N-fragment	91
	**PREDICTED: centromere-associated protein E-like isoform X2**	**gi|721694598**	***B*. *distachyon***	17/42	15	84
**24**	**PREDICTED: 11S globulin seed storage protein 2-like**	**gi|697139898**	***N*. *tomentosiformis***	13/45	64	98
**25**	**PREDICTED: 11S globulin seed storage protein 2-like**	**gi|697139898**	***N*. *tomentosiformis***	13/42	68	115
**26**	**PREDICTED: legumin B-like**	**gi|698538015**	***N*. *sylvestris***	14/41	25 C-fragment	98

These data highlight that, unlike mutant seeds, the proteins needed for the control of mitosis checkpoints were already expressed in the WT dry seeds. Although previously proteomic and genetic analyses have not reported the specific accumulation of proteins involved in the cell cycle in seeds, the presence of the centromere-associated protein E-like isoform X2 (Table 1) in tobacco dry WT seeds suggests that some cell cycle control proteins may be present to enable the rapid elongation of the roots after their protrusion from the micropylar endosperm. This hypothesis is supported by data on barley seeds: cell cycle effectors are present in dry seeds before the cell cycle begins and some embryo cells are stopped in the G2 phase of the cell cycle [54]. In addition, proteins involved in the cell cycle, including also cytoskeleton components, were conserved in *Arabidopsis* dry seeds [50], thus highlighting the important role of these proteins for germination. The lack of the centromere-associated protein E-like isoform X2 in the F18 suggests that some pathways allowing the synthesis of cell cycle proteins may be modified in transgenic tobacco seeds before dehydration. This implies that exogenous DNA insertion may also affect some maturation pathways, such as the synthesis of non-storage proteins (i.e. centromere-associated protein E-like isoform X2 and actin; Tables 1 and 2). The presence of the centromere-associated protein E-like isoform X2 in bands 4 and 5 in WT seeds suggested the presence of a different isoform of this protein. One of these isoforms disappeared from VT2eB seeds. The reason for the delay in root elongation for F18 and VT2eB (Fig 1B) could be due to the need for *de novo* production or the modification of proteins involved in cell cycle progression. Further experiments investigating seed maturation are needed to clarify this hypothesis.

**Table 2:** 2D gel resolved seed polypeptides identified by MALDI-TOF/TOF MS.

Spot N.	Protein description	Accession number	Organism	Mascot search results			Mean % V±SD x 10^−4^^a^		
				N. of matched peptides	Sequence coverage (%)	Score	WT	VT2eB	F18
***Storage proteins***									
**257**	PREDICTED: seed biotin-containing protein SBP65-like isoform X2	gi|1025096692	*N*. *tabacum*	7/14	20	80	-	3091±1484^$^^b^	771±483^$^
**268**	PREDICTED: seed biotin-containing protein SBP65-like isoform X1	gi|1025096688	*N*. *tabacum*	11/19	29	129	140±122*^&^	2300±781*	1428±347^&^
**272**	PREDICTED: seed biotin-containing protein SBP65-like isoform X2	gi|1025096692	*N*. *tabacum*	17/27	39	168	153±133*	4165±1220*	2853±729^&^
**383**	PREDICTED: embryonic protein DC-8-like	gi|1025354957	*N*. *tabacum*	9/13	25	109	-	3835±303	4155±114
**426**	PREDICTED: embryonic protein DC-8-like	gi|1025374019	*N*. *tabacum*	5/5	17	81	-	4201±656	3812±463
**294**	PREDICTED: seed biotin-containing protein SBP65-like isoform X2	gi|698480227	*N*. *sylvestris*	18/13	31	172	-	5246±1718	4238±1551
**763**	PREDICTED: 11S globulin subunit beta-like	gi|697189073	*N*. *tomentosiformis*	16/32	30–N fragment	173	6015±1507*^&^	17341±3788*	15940±2684^&^
**777**	PREDICTED: 11S globulin seed storage protein 2-like	gi|1025029409	*N*. *tabacum*	18/33	46	228	21424±2836*^&^	70003±14299*	63336±1608^&^
**789**	PREDICTED: 11S globulin subunit beta-like	gi|697189071	*N*. *tomentosiformis*	17/50	30 N-fragment	171	3927±382*^&^	18535±1247*	23374±2004^&^
	PREDICTED: legumin B-like	gi|698534493	*N*. *sylvestris*	14/50	29 N-fragment	129			
**790**	PREDICTED: 11S globulin subunit beta-like	gi|1025251419	*N*. *tabacum*	17/36	32 N-fragment	196	4564±244*^&^	19335±5406*	15106±369^&^
**791**	PREDICTED: legumin B-like PREDICTED:	gi|698538015	*N*. *sylvestris*	16/30	26 N-fragment	185	12033±746*^&^	33821±6004*	33199±8730^&^
	11S globulin subunit beta-like	gi|1025258047	*N*. *tabacum*	13/30	20 N-fragment	137			
**806**	PREDICTED: legumin B-like PREDICTED:	gi|1025293602	*N*. *tabacum*	16/41	28 N-fragment	171	5090±572*^&^	34191±7100*	30774±3758^&^
	11S globulin subunit beta-like	gi|697189071	*N*. *tomentosiformis*	13/41	23 N-fragment	122			
**811**	PREDICTED: 11S globulin subunit beta-like	gi|1025251419	*N*. *tabacum*	18/63	33 N-fragment	146	1993±747*^&^	5722±753*	5505±762^&^
	PREDICTED: legumin B-like	gi|698538021	*N*. *sylvestris*	15/63	26 N-fragment	103			
**816**	PREDICTED: legumin B-like	gi|697189071	*N*. *tomentosiformis*	18/41	30 N-fragment	187	2716±427*^&^	7565±564*^$^	9658±1084^&$^
	PREDICTED: 11S globulin subunit beta-like	gi|698534493	*N*. *sylvestris*	16/41	30 N-fragment	156			
**828**	PREDICTED: 11S globulin subunit beta-like	gi|697189071	*N*. *tomentosiformis*	17/37	32 N-fragment	178	1954±41*^&^	9256±1498*	9369±1131^&^
	PREDICTED: legumin B-like	gi|698534493	*N*. *sylvestris*	14/37	30 N-fragment	133			
**838**	PREDICTED: legumin B-like	gi|1025293602	*N*. *tabacum*	17/33	33 N-fragment	194	2914±262*^&^	9349±1096*	8793±1982^&^
**840**	PREDICTED: legumin B-like	gi|698534493	*N*. *sylvestris*	17/49	29 N-fragment	158	1549±468^&^	2447±221	3277±868^&^
	PREDICTED: 11S globulin subunit beta-like	gi|697189071	*N*. *tomentosiformis*	14/49	28 N-fragment	117			
**848**	PREDICTED: legumin A-like	gi|697139891	*N*. *tomentosiformis*	17/39	32 N-fragment	155	9044±1245*^&^	18285±2482*	16861±1198^&^
**850**	PREDICTED: legumin A-like	gi|697139896	*N*. *tomentosiformis*	20/33	40 N-fragment	226	8934±635*^&^	18326±712*	23113±6615^&^
**872**	PREDICTED: legumin A-like	gi|697139891	*N*. *tomentosiformis*	11/22	24 N-fragment	124	799±124^&^	1210±162	2081±683^&^
**880**	PREDICTED: 11S globulin subunit beta-like	gi|697189073	*N*. *tomentosiformis*	7/10	13 Central fragment	81	6154±1607*^&^	402±158*	443±87^&^
**1157**	PREDICTED: legumin A-like	gi|697139896	*N*. *tomentosiformis*	7/9	19	102	2116±838*^&^	422±126*	136±235^&^
**1171**	PREDICTED: legumin A-like	gi|697139891	*N*. *tomentosiformis*	5/5	11 Central fragment	83	1856±212*^&^	391±161*	428±176^&^
**1095**	PREDICTED: vicilin-like antimicrobial peptides 2–3	gi|1025308817	*N*. *tabacum*	8/8	8 Central fragment	108	16436±512*^&^	1139±333*	989±462^&^
**1273**	PREDICTED: legumin A-like	gi|698517368	*N*. *sylvestris*	8/11	20 Central fragment	115	4248±330*^&^	796±437*	423±80^&^
**1357**	PREDICTED: 11S globulin subunit beta-like	gi|697189071	*N*. *tomentosiformis*	11/16	17 Central fragment	139	2462±442*^&^	740±176*	1055±185^&^
**1468**	PREDICTED: 11S globulin subunit beta-like	gi|697189073	*N*. *tomentosiformis*	7/11	13 Central fragment	79	-	667±447	1089±241
***Chaperone proteins***									
**278**	PREDICTED: heat shock 70 kDa protein, mitochondrial	gi|1025062529	*N*. *tabacum*	10/13	17	110	207±196^+^	1006±278^+^	790±484
**1130**	PREDICTED: 17.1 kDa class II heat shock protein-like	gi|1025386162	*N*. *tabacum*	9/14	64	157	5317±328*^&^	13584±3145*	13999±4152^&^
**1133**	PREDICTED: 17.1 kDa class II heat shock protein-like	gi|698539535	*N*. *sylvestris*	8/14	61	116	2933±181*^&^	7652±1605*	8485±2818^&^
**1324**	16.9 kDa class I heat shock protein 1-like	gi|1025247079	*N*. *tabacum*	7/17	37	103		614±167	815±308
**353**	PREDICTED: protein disulfide-isomerase-like	gi|698574414	*N*. *sylvestris*	7/8	15	104	266±275*	1333±197*	845±299
***LEA proteins***									
**713**	PREDICTED: late embryogenesis abundant protein D-34-like	gi|1025097779	*N*. *tabacum*	9/15	37	124	191±51*^&^	1365±418*	1023±132^&^
**792**	PREDICTED: late embryogenesis abundant protein D-34-like	gi|1025079598	*N*. *tabacum*	8/16	43	106	142±124*	1260±523*	883±78
**812**	PREDICTED: late embryogenesis abundant protein D-34-like	gi|1025097783	*N*. *tabacum*	5/12	40	73	149±69*^&^	780±285*	661±84^&^
**837**	PREDICTED: late embryogenesis abundant protein D-34-like	gi|1025073492	*N*. *tabacum*	9/12	47	102	173±41*^&^	898±280*	855±200^&^
***Enzymes***									
**0,7**	PREDICTED: enolase-like	gi|697116359	*N*. *tomentosiformis*	14/16	39	223	290±97*^&^	2092±424*	2427±480^&^
**462**	PREDICTED: enolase-like	gi|697116359	*N*. *tomentosiformis*	6/6	18	101	484±208^£^	1647±1484	1940±484^£^
**959**	PREDICTED: aspartic proteinase	gi|698433659	*N*. *sylvestris*	7/10	14 central fragment	93		415±105	458±75
**696**	PREDICTED: glucose and ribitol dehydrogenase homolog 1	gi|698551643	*N*. *sylvestris*	5/5	16	90		3197±600	3275±890
**730**	PREDICTED: glucose and ribitol dehydrogenase homolog 1	gi|698563269	*N*. *sylvestris*	9/10	28	153	1319±304*^&^	4750±619*	4526±779^&^
**1100**	PREDICTED: methionyl-tRNA formyltransferase-like isoform X3	gi|1025077239	*N*. *tabacum*	5/6	15	84	1664±99^+^	3515±390^+^	5265±2801
***Other*s**									
**0,7**	PREDICTED: actin-97	gi|698564562	*N*. *sylvestris*	18/29	54	206	1180±280*^&^	2738±193*	2602±751^&^
**934**	PREDICTED: uncharacterized protein LOC104224147	gi|698568389	*N*. *sylvestris*	18/42	75	190	3888±543*^&^	8829±824*	10434±2910^&^
**1366**	PREDICTED: MLP-like protein 423	gi|697098884	*N*. *tomentosiformis*	7/11	13 central fragment	79	6624±381*^&^	8694±3214*	6701±4613^&^

^a)^ Each value represents the mean±SD of individually computed %V in spot maps from wild-type (WT), VT2eB-N and F18-N. tabacum dry seeds.

^b)^ Pair-wise comparison was performed using a two-tailed Student’s t-test (p≤0.05) and the Tukey’s post hoc test (p≤0.05). Only protein spots showing both statistical reliability and at least 2 fold change in expression are listed as significant differences: WT vsVT2eB-N(*),WT vs F18-N(&),VT2eB-NvsF18-N($). Significant differences according Student’s t-test between WT and Vt2eB-N, and WT and F18-N are visualized by (+) and (£), respectively

Concerning the storage proteins, bands 8–10 comprise three classes of reserve proteins belonging to vicilin, legumin and globulin families and were only observed in the WT seeds. This suggests that the limited proteolysis leading to the destabilization of the tertiary structures and to the susceptibility of storage proteins to unlimited proteolysis occurred in the WT during seed maturation, whereas it was not observed in the transgenic lines in two independent experiments. In fact, the same proteins were identified in the corresponding bands 13–14 and 22–23, at a higher molecular weight, of the F18 and VT2eB mutants, respectively (Table 1 and Fig 5). The correct folding and packaging of storage proteins play a crucial role in regulating their resistance to proteolysis by specific enzymes [38]. The limited proteolysis of prolegumins in developing seeds as well as that of legumin and vicilin during the seedling development depends on the presence of accessible sites for proteolysis [55]. The limited cleavage destabilizes the tertiary structure of storage proteins and makes them susceptible to further unlimited proteolysis during seed germination and seedling [55]. In addition, the limited proteolysis of storage proteins in WT dry seeds could reflect differences in reserve protein folding and may facilitate the further accessibility of processing enzymes to mobilize the resources, leading to a faster seed germination.

Mass spectrometry analysis of 2D gel resolved polypeptides, performed on dry seeds, confirmed the data obtained by protein identification from 1D gel electrophoresis. In fact, most polypeptides excised from 2D gels, which differed in the transgenic seeds with respect to the WT, were reserve proteins belonging to the vicilin, globulin and legumin protein families (Table 2). A 2D gel image analysis showed that a number of spots identified as storage proteins were significantly more abundant in F18 dry seeds, compared with the WT (compare Figs 6 and 7, black bold spots). The number of enhanced reserve proteins also significantly increased in the VT2eB seeds, with respect to the WT (compare Figs 6 and 8, black bold spots). These data confirm that the delay in the germination time of VT2eB and F18 seeds could be correlated to the amount of storage proteins and/or to their folding/assembly state [38].

Although previous studies have suggested that there is no relationship between total protein content and germination rate, the authors do not exclude the possible relationship between germination and specific classes of seed proteins [47].

Our data support a correlation between the increase/folding state of reserve proteins both with delayed germination and with the persistent seed profile. The higher storage protein content for buried seeds could be to support the higher need for nutrients of embryos that need to grow for a longer time until the seedling reaches the soil surface and initiates photosynthesis. In addition, buried seeds showed a round shape and a delayed germination [46, 47] like the transgenic line seeds.

2D gel analysis also confirmed that the proteolysis of storage proteins (legumin, globulin, vicilin) occurred in WT dry seeds, since polypeptides which are shown in black in Figs 7 and 8 with a low molecular weight, were significantly higher in WT seeds with respect to the transgenic lines. These differences suggested that the proteolysis of storage proteins during seed development was poor in the VT2eB and F18 seeds, explaining the retarded germination of mutant seeds.

Interestingly, in addition to the storage proteins, five spots were identified as chaperone proteins with a different molecular mass and belonging to Hsp70, small Hsp proteins (sHsp) and protein disulfide isomerase families (Table 2; Figs 7 and 8, green spots). Two were significantly enhanced only in VT2eB dry seeds, compared with the WT (Table 2; Fig 8, green spots in bold), while sHsp were also significantly enhanced in F18 (Table 2; Fig 7, green spots in bold). One spot identified as 16.9 kDa class I heat shock protein 1-like was detected only in mutant seeds (Table 2; Figs 7 and 8, green spot 1324). A high range of proteins were found to have chaperone activity. These included many proteins that were identified as heat shock proteins (Hsp), while others were identified as protein disulfide isomerases [56, 57]. Hsps and chaperones are considered as the major class of stress responsive proteins, involved in decreasing cellular damage following abiotic stresses [58, 59]. Hsps/chaperones can be localized in the cytoplasm or in membranous organelles where they assist protein folding and transport in control and in stress conditions [60]. They are involved in a wide range of stress responses such as cold, heat, drought and oxidative stress.

Thirteen sHsps were identified in the *Arabidopsis* genome and classified into six classes, depending on the subcellular localization and sequence homology [61], highlighting the high capacity of plants to deal with stress adaptation [62]. In addition, the endoplasmic reticulum Hsp70 (Bip) also regulates the protein trafficking to the Golgi apparatus before the further sorting to the PM or the vacuole. Bip also assists and facilitates protein folding and assembly [63] and may have a crucial role in assisting and regulating the appropriate folding of storage proteins during seed development. In *Arabidopsis*, Hsp70 was observed at high levels in after-ripening non dormant seeds and is required during dormancy release to maintain the correct folding of other proteins [50].

Among the chaperone proteins, a protein disulfide isomerase spot was also significantly enhanced in VT2eB, compared to the WT (Table 2). This protein showed an active thioredoxin-like domain and an ER resident signal and was involved in introducing disulfide bonds to nascent polypeptides in the ER lumen [64]. In seeds, the protein disulfide isomerase was found to play a different role related to protein folding (Kim et al, 2012; Kimura et al., 2015), regulation of cysteine protease activity [65], chaperone activity [66, 67], promotion of specific localization of Cys-rich prolaminin in the core of PBs [68] and regulation of the proportion of various seed proteins, including storage proteins [69]. In wheat, disulfide isomerase protein play an important role in assisting the folding of newly synthetized proteins during germination and in forming disulfide bonds in seed storage proteins [70].

Both Hsp70 and protein disulfide isomerase control protein folding thereby stabilizing their structure. The increase in these chaperon proteins may go hand in hand with the increase in storage proteins observed in tobacco VT2eB seeds with respect to WT. It is possible that the increase in these proteins in transgenic seeds was insufficient for the correct folding of storage proteins and thus for the correct mobilization of storage material for embryo development. In F18 seeds, the increase in storage proteins was not accompanied by the enhancement of these chaperone proteins (Hsp70, protein disulfide isomerase), thus explaining the higher germination delay observed in this transgenic line (Fig 1).

Interestingly, in wheat seeds, proteomic and mRNA analyses showed that the repression of disulfide isomerase in after-ripening compared to dry seeds, promotes proteolysis and in turn seed dormancy release and germination [71]. It is possible that the increase in this protein in transgenic lines contributes to germination delay.

In VT2eB seeds, the number of stress-related proteins such as late embryogenesis abundant (LEA) proteins and sHSPs (Table 2; Fig 8, violet/green bold spots), was also significantly higher than in WT seeds. These proteins also increased significantly in F18 seeds although to a lesser extent with respect to VT2eB (Table 2; Fig 7, violet/green bold spots). LEA genes are expressed during the later stage of seed maturation and are involved in the acquisition of desiccation tolerance [72]. It has also been proposed that LEA proteins, which are localized in the nuclei, may have enzymatic or chaperone activity in nucleus proteins that unwind or repair DNA, regulate transcription, and might be associated with chromatin or cytoskeleton [73].

The LEA-like proteins, which increased in VT2eB and F18, belong to Group 5 which includes atypical LEA proteins with a significantly higher proportion of hydrophobic residues [74–76]. Group 5 LEA proteins are also expressed in seeds during the late maturation stage of development [77]. Unlike other groups of LEA proteins, which show high hydrophilic residues and play a role in protein protection from desiccation, very few studies have characterized group 5 LEA functions in abiotic stress tolerance. This LEA group could be involved in membrane protection [78]. This protective effect has also been observed in tobacco seeds overexpressing a novel atypical group 5 LEA gene from *A*. *diogoi* (AdLEA). This protein plays a role in abiotic stress tolerance, most specifically in water limiting conditions by increasing O_2_-scavenging and up-regulating various stress-related genes [79].

The abundance of these proteins in transgenic lines could be due to the necessity to protect membranes and storage lipids from desiccation and to defend them from ROS activity during after-ripening and early germination. This characteristic could be related with persistent trait of buried seeds, which remain longer in the soil and are more subjected to oxidative damages.

In F18, the increase in these proteins was lower than VT2eB and, in particular, spot 792, identified as a LEA protein, was not significantly different from WT. This difference could further explain the higher delay in F18 seed germination compared to WT and VT2eB seeds.

Therefore, in F18 seeds, the increase in storage proteins was not accompanied by a parallel increase in chaperone proteins (Table 2), thus favoring protein oxidative stress and aggregate formation during dehydration, thereby resulting in an inability to use seed storage material for germination.

Interestingly, LEA proteins are also considered important for the persistence of buried seeds in a natural environment since they facilitate intracellular ‘glass formation’ in dehydrated cells [80, 81] inducing low metabolic activity and facilitating the persistence of dry seeds in the soil. The higher increase in LEA proteins represents an additional trait that, together with the shape change (Fig 2F), correlate the behavior of the transgenic lines with that of persistent buried seeds. This data suggests that the relative number of storage proteins and of proteins regulating their folding/accumulation state represent common mechanisms to control seed germination and that the destiny of seeds is already determined during maturation.

In addition to LEA proteins, sHsps also have an overall protective effect during seed drying. In both transgenic lines, the number of two sHSPs belonging to Class I (16.9 kDa class I heat shock protein 1-like) and II (17.1 kDa class II heat shock protein-like), increased in dry seeds, as detected by 2D gel analyses (Table 2; Fig 8, green spot 1130, 1133, 1324). sHsps might act as molecular chaperones during seed dehydration and during the first few days of rehydration. In seeds, class I and class II sHsps are developmentally regulated: they accumulate during seed maturation, before the acquisition of desiccation tolerance [82], and disappear in parallel to storage protein degradation [83]. These proteins stabilize protein conformation and help in protein folding, oligomer formation, intracellular transportation, and marking for degradation [78, 84, 85]. As observed for LEA proteins, sHsps may be required for desiccation tolerance [72, 86], and it has been observed that, in *Synechocystis*, Hsp17 could play an important role in membrane quality control and in the maintenance of membrane integrity [87]. In transgenic seeds, a 17.1 kDa class II heat shock-like protein significantly increased with respect to WT in dry seeds (Table 2). This protein has not yet been characterized and may participate in the protection of proteins or the membrane during seed desiccation.

Interestingly, spot 1324 identified as 16.9 kDa class I heat shock protein 1-like was only detected in mutant seeds and not in WT. In rice, Oshsp16.9 gene is expressed during stress responses and transgenic plants have shown tolerance to salt, cold, heat and dehydration stresses [88, 89]. As observed with other stress response proteins which increase in transgenic lines in parallel with storage proteins, it is possible that these proteins play a role in keeping proteins in a folding-competent state during seed desiccation and in preventing them from irreversible aggregation until ATP-dependent chaperones (such as Hsp70 and Hsp60 GroE) restore the refolding of denatured proteins to native physiological conditions [90]. In this way, storage proteins become accessible to degradation during germination. In summary, the delay in transgenic seed germination was probably due to the increasing number of storage proteins which was associated with the higher persistence of seeds in the natural environment. In the VT2eB line, the increase in storage material was accompanied with an increase in chaperone and stress related proteins. However, the increase in chaperone proteins in parallel with storage proteins appeared insufficient for the correct germination of transgenic seeds. In the F18 line, the increase in storage proteins was only partially accompanied by an increase in chaperone proteins so that the storage proteins did not fold correctly for proteolysis, further delaying early germination events.

Notably, sHsp, which increase in F18 seeds (Table 2), are not able by themselves to determine the protein folding but they bind and stabilize proteins to prevent their possible non-native aggregation, facilitating subsequent refolding by other chaperones such as Hsp70 [91, 92]. Therefore, Hsp70s, which in turn did not increase significantly in F18 seeds (Table 2), interconnect with other chaperones to form the chaperone cell network and are also involved in responding to environmental stimuli [93]. Therefore, the higher delay in the germination of F18 seeds with respect to WT and VT2eB may also be due to the loss of cooperation between sHsp and Hsp70 in the protein folding activity.

In addition to storage material and chaperone proteins, F18 and VT2eB showed significant alteration in the enzymes involved in amino acid, lipid and sucrose metabolisms (Table 2; Figs 7 and 8, blue bold spots). Enolase appeared significantly increased in both seed mutants with respect to WT although to a greater extent in VT2eB transgenic line (Table 2; Figs 7 and 8). This enzyme is involved in glycolysis. It catalyzes the reversible dehydration of 2-phosphoglycerate (2PGA) to phosphoenolpyruvate (PEP) and plays an important role during adaptation to anaerobiosis [94]. PEP generated through the enolase reaction in the cytosol is also a central metabolite in plant primary and secondary metabolism. It is involved in the tricarboxylic acid (TCA) cycle occurring into the plastid stroma, and acts as a precursor for the biosynthesis of aromatic amino acids in the shikimate pathway and for the biosynthesis of fatty acids [95–97], branched chain amino acids [98] and isoprenoids [99]. The alteration in the carbohydrate and lipid metabolism could affect seed germination in F18 and VT2eB. It is known the carbohydrate content controls the entry of water into the seed during imbibition [47]; therefore the modification of sugar metabolism could affect imbibition thus contributing to the delay in the storage material mobilization observed in transgenic comparing to WT seeds in tobacco.

In addition, glucose and ribitol dehydrogenase homologs 1 (GRDs) are involved in the carbohydrate metabolism and increased in transgenic with respect to WT seeds (Table 2; Figs 7 and 8). An increase in the expression of GRD was observed in seeds and tissues after heat, salinity and anoxic stresses, suggesting a role in the accumulation of sugars with an osmo-protective function [100–103]. The increase of these proteins in transgenic lines of tobacco (F18, VT2eB) could interfere with the carbohydrate metabolism and thus with the water uptake during imbibition, thereby inducing a delay in the reserve mobilization observed by morphological analyses. In addition, proteomic analyses of dry and after-ripening wheat seeds showed that imbibition of after-ripening seeds led to a substantial repression of glucose/ribitol dehydrogenase compared to dry seeds, thus suggesting that suppression of GRDs could be related to germination [71]. The presence of high GRD content in F18 and VT2eB with respect to WT tobacco seeds could contribute to the delay in transgenic seed germination.

Aspartic proteinase was only detected in transgenic seeds (Table 2; Figs 7 and 8). Aspartic proteinase was involved in the proteolytic processes of storage proteins during seed maturation and participates in the mobilization of storage proteins during seed germination [104–107]. In *Arabidopsis* seeds, these enzymes colocalize in the PBs with the seed storage protein 2S albumin and the vacuolar marker α-mannosidase [108]. In addition, in *Arabidopsis* seeds, proteolytic processing of 2S albumins occurs inside multivesicular bodies (MVBs) before the storage proteins reach the PBs. Golgi-derived vesicles carrying aspartic protease are different from vesicles carrying storage proteins. These vesicles fuse with the same MVBs where proteolysis of 2S albumins occurs [109]. The presence of aspartic proteinase only in tobacco F18 and VT2eB seeds suggests that the maturation process leading to the proteolysis of storage proteins had not been completed in the transgenic seeds and that this enzyme was still present in dry seeds. This is in line with the absence of limited proteolysis observed in 1D and 2D gel analyses (Table 2; Fig 6).

Other proteins significantly enhanced in transgenic tobacco seeds, such as the MPL-like protein, Methyonil tRNA formyltransferase and an uncharacterized protein LOC104224147, were less characterized (Table 2; Figs 7 and 8, red bold spots). The MPL-like protein is a low-molecular-weight polypeptide called a major latex protein (MLP) which is abundant in the latex from the opium poppy (*Papaver somniferum*) [110, 111]. This protein was later found in other plants, such as tobacco [112, 113]. The function of MLPs is unknown and they have been associated with fruit and flower development and in pathogen defense responses [114]. The MLPs expression pattern is similar to some of the intracellular pathogenesis-related (IPR) proteins [115]. No relation between the expression of all these proteins and seed germination has been reported, and it is possible that their increase could be related to the response induced by exogenous DNA insertion and exogenous EV protein expression.

## Conclusions

Tobacco transgenic seeds, created by the insertion of DNA codifying EV, showed a different germination and seedling ability compared to the WT, suggesting that exogenous DNA insertion interfered with endogenous protein expression and with germination. Morphological and proteomic analysis revealed new insights into the traits that influence germination. The findings highlight that the assumptions of germination are determined during seed maturation, in terms of storage protein accumulation and processing and of carbohydrate metabolism, which regulates water uptake during the early phases of germination. In addition, morphological and proteomic seed modifications support the theory that seed shape and storage protein content are related to seed dormancy and persistence in soil, which in turn are important in terms of the role of biodiversity and conservation played by seeds.

## Supporting information

S1 Fig Detection of VT2e-B and F18 genes in the transgenic tobacco plants. **A, B** pBIpGLOB binary vectors maps for F18 and VT2eB. **C** DNA samples from WT and transgenic lines were analyzed by PCR using specific primers for the detection of VT2e-B and F18 genes in R3 generation. The analyses confirmed the stable integration of the exogenous genes in both lines of tobacco plants.
(TIF)

S2 Fig Soil seed germination. The graph shows the mean time of seedling of WT and transgenic lines seeds grown in soil. Seedling time was significantly delayed in transgenic seeds compared to WT.
(TIF)
