# Supplementary figures and images for: Retarded germination of Nicotiana tabacum seeds following insertion of exogenous DNA mimics the seed persistent behavior
Source: PLoS One. 2017 Dec 7;12(12):e0187929. doi: 10.1371/journal.pone.0187929 (PMC5720674; doi:10.1371/journal.pone.0187929)

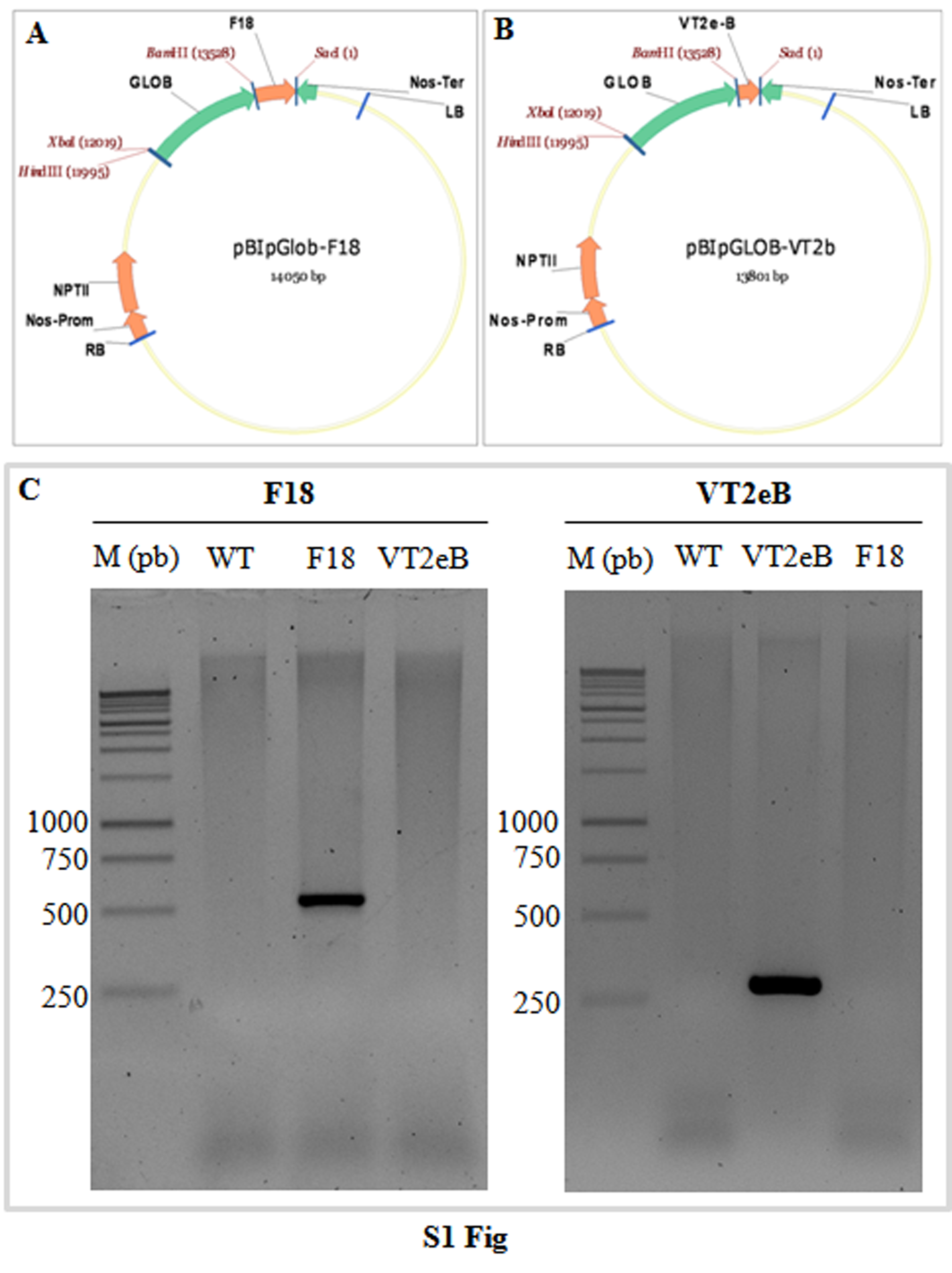

Supplement: S1 Fig — A, B pBIpGLOB binary vectors maps for F18 and VT2eB. C DNA samples from WT and transgenic lines were analyzed by PCR using specific primers for the detection of VT2e-B and F18 genes in R3 generation. The analyses confirmed the stable integration of the exogenous genes in both lines of tobacco plants. (TIF) [file pone.0187929.s001.tif]

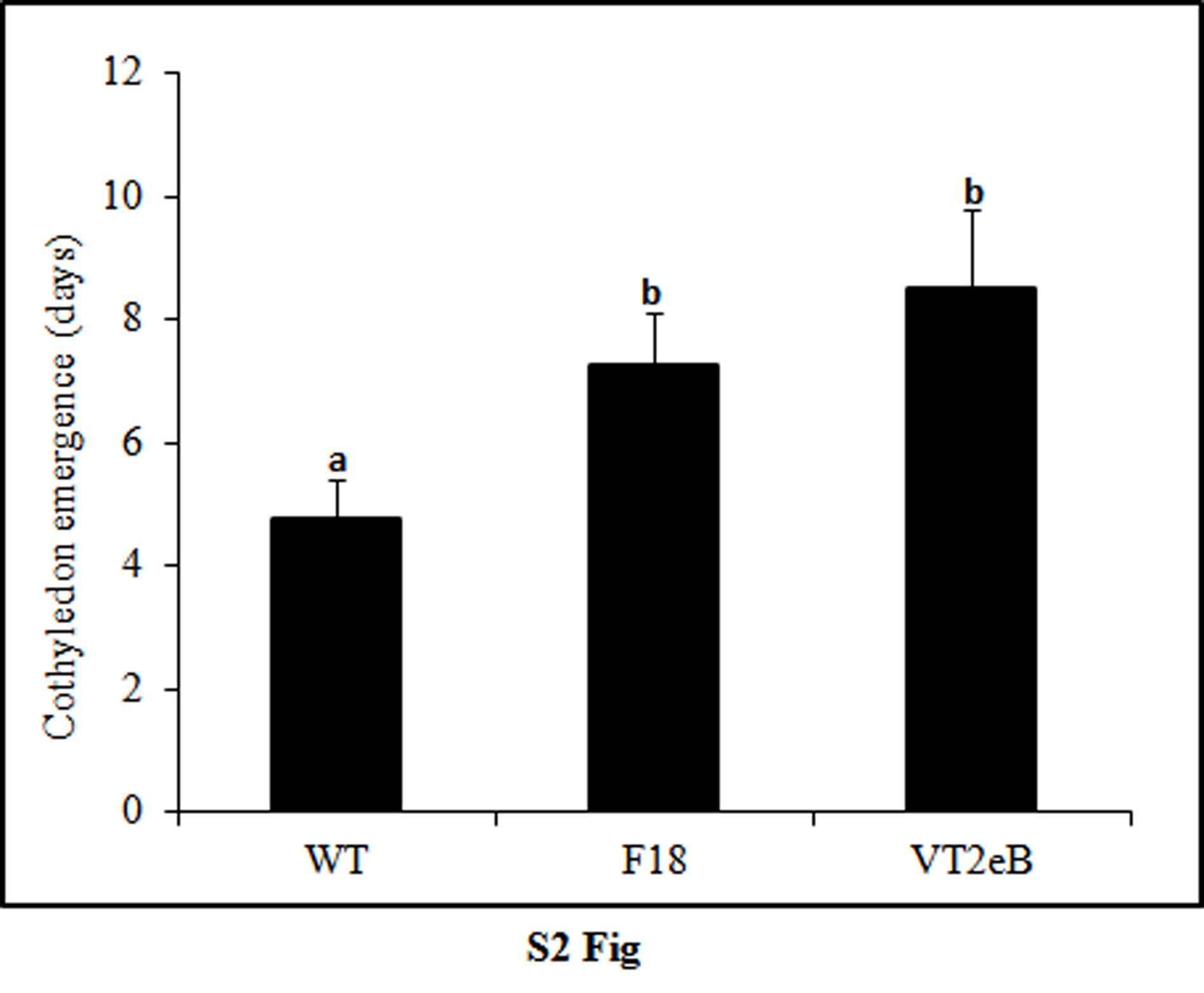

Supplement: S2 Fig — The graph shows the mean time of seedling of WT and transgenic lines seeds grown in soil. Seedling time was significantly delayed in transgenic seeds compared to WT. (TIF) [file pone.0187929.s002.tif]
